# High Strength Titanium with Fibrous Grain for Advanced Bone Regeneration

**DOI:** 10.1002/advs.202207698

**Published:** 2023-04-07

**Authors:** Ruohan Wang, Mingsai Wang, Rongrong Jin, Yanfei Wang, Min Yi, Qinye Li, Juan Li, Kai Zhang, Chenghua Sun, Yu Nie, Chongxiang Huang, Antonios G. Mikos, Xingdong Zhang

**Affiliations:** ^1^ National Engineering Research Centre for Biomaterials/College of Biomedical Engineering Sichuan University Chengdu 610065 China; ^2^ School of Aeronautics and Astronautics Sichuan University Chengdu 610065 China; ^3^ Department of Orthopedics Orthopedic Research Institute West China Hospital Sichuan University Chengdu 610041 China; ^4^ Department of Chemistry and Biotechnology Centre for Translational Atomaterials Swinburne University of Technology Hawthorn VIC 3122 Australia; ^5^ State Key Laboratory of Oral Diseases West China School of Stomatology West China Hospital of Stomatology Sichuan University Chengdu 610041 China; ^6^ Departments of Bioengineering Chemical and Biomolecular Engineering Rice University Houston TX 77251 USA

**Keywords:** anatase titanium oxide, bone regeneration, contact guidance, fibrous grains, high strength, pure titanium

## Abstract

Pure titanium is widely used in clinical implants, but its bioinert properties (poor strength and mediocre effect on bone healing) limit its use under load‐bearing conditions. Modeling on the structure of collagen fibrils and specific nanocrystal plane arrangement of hydroxyapatite in the natural bone, a new type of titanium (Ti) with a highly aligned fibrous‐grained (FG) microstructure is constructed. The improved attributes of FG Ti include high strength (≈950 MPa), outstanding affinity to new bone growth, and tight bone‐implant contact. The bone‐mimicking fibrous grains induce an aligned surface topological structure conducive to forming close contact with osteoblasts and promotes the expression of osteogenic genes. Concurrently, the predominant Ti(0002) crystal plane of FG Ti induces the formation of hydrophilic anatase titanium oxide layers, which accelerate biomineralization. In conclusion, this bioinspired FG Ti not only proves to show mechanical and bone‐regenerative improvements but it also provides a new strategy for the future design of metallic biomaterials.

## Graphical abstract

Inspired by the microstructure of natural bone, a hierarchical fibrous‐grained microstructure with highly crystallographic orientation in pure Ti endows high strength of near gigapascal and outstanding bioactivity for osseointegration. The specific microstructure produces an intrinsically aligned surface topological structure for contact guidance of osteoblasts and osteogenesis promotion and forms a subnanometer hydrophilic anatase TiO_2_ layer for fast biomineralization.

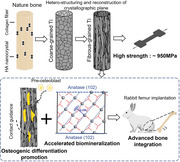

## 1 Introduction

Commercially pure titanium (Ti) is widely used in clinical implants for bone defects because it has acceptable biocompatibility.^[^ ^1^ ^]^ However, its load‐bearing capability is limited by poor strength and mediocre effect on bone healing. Doping pure Ti with additional elements can ameliorate its poor mechanical performance. For this reason, alloyed titanium such as Ti6Al4V remains the primary choice in orthopedics.^[^ ^2^ ^]^ However, alloy implants often carry safety concerns. Leaked vanadium and aluminum from Ti alloys are cytotoxic and prolonged exposure can lead to adverse osteolysis and neural disorders.^[^ ^3^ ^]^ Therefore, a strong and more bio‐friendly form of pure Ti is demanded.

Nature bone is a hierarchically ordered network of collagen fibrils and hydroxyapatite (HA) nanocrystals selected by natural evolution. The *c*‐axis of the HA nanocrystals prefer to orient along the collagen fibrils.^[^ ^4^ ^]^ Besides providing a sturdy mechanical support, these architectures guide the orderly formation of bone. It is suggested that manipulating the microstructure can compensate for the mechanical losses of dealloying. During the past two decades, by applying the technique of severe plastic deformation such as equal channel angular pressing, high pressure torsion, accumulative rolling bonding, etc.,^[^ ^5^ ^]^ pure Ti has already been strengthened to near‐gigapascal levels with the grain size refining to the nanoscale. However, such materials have low ductility and quick fracture after yielding in service.^[^ ^5a,e^ ^]^ The hierarchical architecture of bone, in which soft and hard domains are orderly organized at multiscale levels, provide further inspiration for the development of bone‐compatible materials. For instance, heterogenous domains with dramatic grain‐size difference can be properly deployed to optimize the mechanical properties of pure Ti. A heterogenous lamella structure with ultrafine‐grain strength and coarse‐grained ductility has been already synthesized.^[^ ^6^ ^]^ However, satisfying the biological properties while engineering microstructures with high mechanical performances remains a difficult task.

The collagen of natural bone has a fibrous topology and osteocytes secrete various bioactive substances to promote bone growth or repair. Thus, extensive efforts have focused on fabricating implants with favorable bio‐interfaces. Among the techniques applied are surface engineering of the physical morphology and roughness, coating with bioactive molecules, and adjusting the oxide type and chemical composition of materials.^[^ ^7^ ^]^ Grooved micropatterns‐based topological structures are the most widely used biomimetic method to promote osteogenic differentiation, which were usually sculpted on the surface by laser and reactive ion etching.^[^ ^8^ ^]^ Aligned microgrooves can induce the cytoskeleton rearrangement followed by downstream signal transduction, thus resulting in directional adhesion and migration of osteoblasts as well as improved osseointegration process.^[^ ^9^ ^]^ Many researchers modify the surfaces of metal implants with bioactive molecules (such as HA, miR‐21, BMP‐2, and BMP‐4) to accelerate cellular attachment, proliferation, and extracellular matrix synthesis.^[^ ^10^ ^]^ Meanwhile, anatase grown by anodization on Ti presents better adhesion, propagation, and differentiation of the osteoblasts than rutile titanium dioxide (TiO_2_) on Ti.^[^ ^11^ ^]^ Cell attachment or biomineralization of Ti‐based substrates is likely to crystallographically texture‐sensitive.^[^ ^12^ ^]^

In the above reports, the bone‐mimicking microstructure enhanced either the mechanical strength or bioactivity. Can we find a Ti microstructure that simultaneously improves the mechanical and biological properties? As a proof‐of‐concept, we describe a novel hierarchical fibrous‐grained (FG) microstructure in pure Ti. The specific fibrous microstructure with a preferred texture produces an intrinsically oriented microgrooved surface and a subnanometer hydrophilic anatase TiO_2_ layer, ensuring fast osseointegration and remarkably bone‐regeneration ability. Importantly, the hierarchical FG microstructure can be fabricated by conventional extrusion and a rotary swaging process, thus can be economically scaled up for industrial production.

## 2 Results and Discussion

### 2.1 Hierarchical Fibrous‐Grained Microstructure

**Figure 1:**
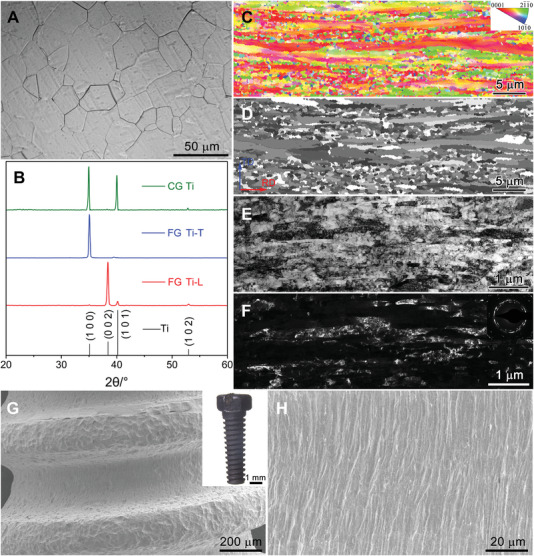
Microstructure of FG Ti. A) Optical image of equiaxed CG microstructure in initial Ti sample. B) XRD spectra of the FG Ti‐L, FG Ti‐T, and CG samples. C) Typical longitudinal sectional (TD–RD plane) EBSD inverse pole map, showing the highly textured fiber‐like hierarchical microstructure. D) Typical EBSD image showing the fiber‐like hierarchical microstructure (TD–RD plane). E,F) Typical longitudinal‐sectional bright‐field and dark‐field TEM images showing the ultrafine fibrous microstructure. G) SEM image showing the surface morphology of the FG Ti screw implant after surface treatment (sandblasting and acid etching). The inset is the screw implant. H) High‐magnification observation of the implant surface showing its typical fibrous morphology.

To achieve a bio‐friendly microstructure with the mechanical properties of pure Ti, we build a refined bone‐like hierarchical microstructure and tune its crystal orientation. Specifically, we sculpt ultrafine fibrous grains embedded with equiaxed nanograins (**Figure** **1**A–F). Starting from pure Ti rods (Grade 2 in the American Society for Testing and Materials (ASTM); see Table S1, Supporting Information), with coarse‐grained (CG) microstructures (Figure 1A), we elongated the initial large equiaxed grains into many thin and long forms using hot extrusion and cold rotary swaging. The elongated forms were observed in the longitudinal‐sectional plane using electron back‐scattered diffraction (EBSD, Figure 1C). To tailor the microstructural heterogeneity, the material was further annealed at its critical recrystallization temperature. During the annealing step, embedded nanograins were formed by partial microstructural recovery, as verified by EBSD and transmission electron microscope (TEM) observations (Figure 1D–F and Figure S1A,B, Supporting Information). Cross‐sectional TEM and EBSD observations show nearly equiaxed ultrafine grains (Figure S1C–E, Supporting Information), confirming that the elongated grains viewed on the longitudinal‐sectional plane are fiber‐like grains. The fibrous grains are 182 ± 26 nm in diameter and several tens of micrometers long. They are characterized by a notably strong texture and their Ti(0002) plane lies parallel to the rod axis (Figure 1B,C and Figures S1F and S2, Supporting Information), i.e., their basal planes face outside. When the FG Ti was machined into a screw and further treated by sandblasting and acid etching, together with a disk with acid etching or electrolytic polishing, the surface morphology displayed a typical fiber‐like structure (Figure 1G,H and Figure S3, Supporting Information). Such a specific morphology is acquired through the thermal‐mechanical treatment in the fabrication process and completely different from the artificial grooves conventionally sculpted by laser interference lithography and reactive ion etching.^[^ ^13^ ^]^ The highly textured fibrous grains substantially reinforce the material and provide unexpected bioactivity for bone growth.

### 2.2 Improved Mechanical Properties

**Figure 2:**
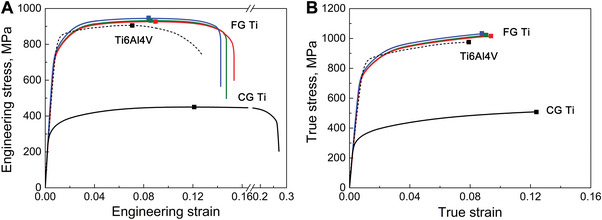
Uniaxial tensile behaviors of the FG Ti. A) Typical engineering stress–strain curves of FG Ti. The curves of CG Ti, and Ti6Al4V alloy are provided for comparison. B) Comparison of true stress–strain curves, showing the higher strain hardening capability of FG Ti than that of the CG counterpart and Ti6Al4V samples.

Uniaxial tension tests reveal good mechanical behaviors of FG Ti along the rolling direction (RD, **Figure** **2**A). The ultimate tensile strength reached 946 ± 14 MPa and the elongation‐to‐fracture was 14.6 ± 1.0% (Table S2, Supporting Information), superior to those of Ti6Al4V alloy (International Organization for Standardization 5832–3 and ASTM 1472‐14) and ultrafine‐grained Ti strengthened by simply reducing grain size (Figure S4 and Table S3, Supporting Information). The strain‐hardening ability of the FG Ti sample was especially strong (Figure 2B). The strain hardening exponent of FG Ti is as high as 0.124, approaching that of CG Ti (0.144) and surpassing that of Ti6Al4V alloy (Figure S5, Supporting Information). The unexpectedly high strain‐hardening rate imparts a large uniform elongation to FG Ti (8.2% ± 0.6%, exceeding that of Ti6Al4V) (Figure 2A). In the hierarchical microstructure, such high strength can originate from the refined grains and microstructural heterogeneity; the former enhances the grain‐refinement strengthening and the latter acquires synergetic strengthening/hardening from interactions among the deformation‐incompatible heterogenous constituents.^[^ ^14^ ^]^ Meanwhile, the high strain hardening and ductility are attributed to i) accumulation of extra geometrically necessary dislocations among synergetic deformation of the hierarchical microstructure and ii) the notably strong texture which is suitable for the activation of prismatic <a> slip.^[^ ^14d^ ^]^

### 2.3 Osseointegration Capability

**Figure 3:**
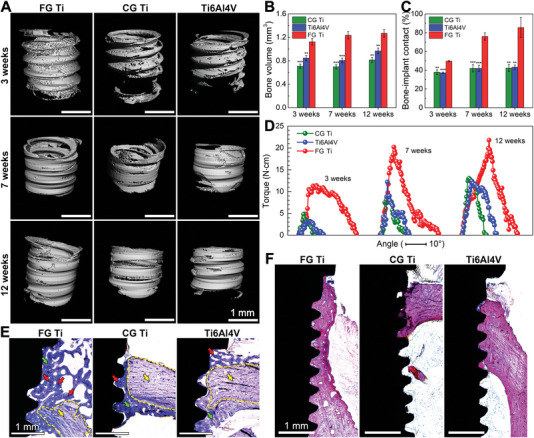
Osseointegration effect of FG Ti, CG Ti, and Ti6Al4V after implantation. A) 3D reconstruction models based on micro‐CT images of newly formed bone in the holes drilled for implantation. B) Volumes of newly formed bone calculated from the 3D reconstruction models. C) Bone‐implant contact percentage of the samples after implantation at different times. **p < 0.01, ***p < 0.001 versus FG Ti. D) Torque‐angle curves of various implants after removal from the integration interface by a digital torque wrench. Optical microscope images of E) bone tissue stained with toluidine blue around the implants at 3 weeks and F) bone tissue stained with basic fuchsin around the implants at 12 weeks. Yellow dotted line: border of old bone, green arrows: new bone, yellow arrows: old bone, red arrows: bone lacuna.

To test the bone‐regeneration efficacy of the FG Ti, we machined rods of FG Ti, CG Ti, and Ti6Al4V into screws for implantation in rabbit femurs (Figures S6 and S7A–E, Supporting Information). New bone formation around the implants was visualized through micro‐computed tomography (micro‐CT)‐based 3D image reconstruction (**Figure** **3**A). During the early stage (3 weeks), the average bone volume was already larger around FG Ti (1.12 mm^3^) than around CG Ti and Ti6Al4V at 12 weeks (0.81 and 0.97 mm^3^, respectively; see Figure 3B). This result indicates a shorter and earlier healing process of the FG Ti implant than of the other implants. The trabecula numbers and trabecula separations did not significantly differ among groups, indicating a normal structure of new bone (Figure S7F, Supporting Information). Furthermore, the bone‐implant contact of FG Ti (85.58%) was approximately twice that of CG Ti (43.03%) and Ti6Al4V (43.21%) at 12 weeks (Figure 3C). In general, such fast osseointegration can only be achieved by implant surface modification with genes or peptides, such as miR‐21, cellular adhesive, and osteogenetic peptides.^[^ ^10^, ^15^ ^]^

Torque tests also demonstrated the superior biomechanical evaluation of FG Ti. The torque force and angle, which together determine the work done by twisting the implant, might also reflect the binding capacity between the implants and bone tissues. The maximum torque of FG Ti at 3 weeks (11.7 Ncm, torque angle = 28.67°) was already comparable to those of CG Ti (13.1 Ncm, 13.33°) and Ti6Al4V (12.4 Ncm, 14.33°) at 12 weeks (Figure 3D and Figure S7D, Supporting Information).

The superior bone‐integration properties of FG Ti are probably caused by rapid deposition of new bone, which increases the bioactive bonding between the implants and bone tissues. To clarify this proposition, the early‐stage (3 weeks) capacity of bone regeneration was histologically evaluated by toluidine blue and calcein staining (Figure 3E and Figure S8A,B, Supporting Information). A large amount of newly formed trabecular bone (green arrows) appears around the old bone (yellow arrows, boundary marked by the yellow dotted line) at the interface of FG Ti/bone, whereas little bone growth appears around the interfaces of CG Ti/bone and Ti6Al4V/bone (Figure 3E). The FG Ti group also shows obvious bone lacunae (red arrows) in the trabecular bone, which are scarce in the CG Ti and Ti6Al4V samples. Consistent with the micro‐CT results (Figure 3A), the sections of bone tissue stained with basic fuchsin, which confirm the final bone formation (Figure 3F and Figure S8C, Supporting Information), reveal more threads covered by new bone in the FG Ti group than in the other materials.

Micro‐CT and histological evaluation in vivo showed that FG Ti induces a larger amount of new bone formation at faster rate than the control materials. The related micro‐CT and biomechanics parameters of FG Ti at 3 weeks almost matched those of the control groups at 12 weeks. At 12 weeks, the bone to implant contact (BIC) of FG Ti was twice those of CG Ti and Ti6Al4V, exceeding those of reported implants coated with bioactive molecules such as miR‐21,^[^ ^10c^ ^]^ nano‐HA,^[^ ^16^ ^]^ and dual‐functionalized peptides of osteogenic growth factor (DOPA)_4_‐S_5_‐GRGDS (RGD peptide) together with (DOPA)_4_‐G_4_‐YGFGG.^[^ ^17^ ^]^

### 2.4 Contact Guidance of Osteoblasts and Osteogenesis Promotion

Aligned microgrooves stimulate the directional adhesion and migration of osteoblasts,^[^ ^18^ ^]^ which benefits the contact guidance and osseointegration process.^[^ ^19^ ^]^ Thus, we explored the osteoblast behavior and osteogenesis‐related gene expression on the surface of FG Ti sectioned along the longitudinal direction of the rods. Focal adhesions (FAs) are plasma membrane‐associated macromolecular assemblies that interact with the surrounding extracellular matrix via integrin receptors, thus determining the spreading and mechanotransduction of cells. In the intracellular FA vinculin, the tail domain binds to F‐actin and paxillin. Therefore, by visualizing vinculin, we can observe the formation of FAs on varied surfaces and the anchoring of F‐actin to the cell membrane.

**Figure 4:**
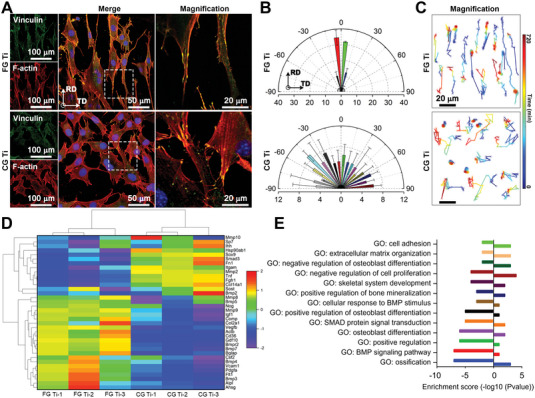
Osteogenesis promotion of oriented grains on FG Ti and CG Ti. A) CLSM images of vinculin (green) and F‐actin (red) immunofluorescence‐stained MC3T3‐E1 cells. Nuclei were marked with DAPI (blue). B) Cell‐alignment observation and analysis on FG Ti and CG Ti after acid etching. The FG Ti samples were sectioned along the longitudinal direction of the rods. Polar plots of cell appearance: frequency distribution (%) and angles (°) between the long axis of the MC3T3‐E1 cells and Ti‐grain orientations. The cells align along the RD on FG Ti and in arbitrary direction on CG Ti. C) Migration trajectory of the MC3T3‐E1 cell, obtained in a live‐cell imaging system monitored for 12 h with 5 min interval photographing. D,E) Hierarchical clustering and GO function analysis based on osteogenesis‐related gene expression data from MC3T3‐E1 cells culture after 7 days of osteogenesis induction with the osteogenic medium. In (E), positive values represent the upregulated gene numbers of cells on CG Ti compared to FG Ti, and negative values represent the upregulated gene numbers of cells on FG Ti compared to CG Ti.

When spread on the Ti surface, the FA complex of osteoblast progenitor cells (MC3T3E‐1) was parallelly anchored on the oriented cellular stress fiber along the RD of FG Ti. The FA complex on the FG Ti sample showed a narrow morphology, typically aggregating on one ridge and the neighboring fibrous grains (**Figure** **4**A). Next, more than 200 of the MC3T3E‐1 cells were randomly selected for statistical analysis. Most of the cells (67.30%) on the FG Ti sample spread along the elongated grains with an angular range of −5° to 5°, whereas only 11.67% of the cells exhibited this behavior on CG Ti (Figure 4B and Figure S9A,B, Supporting Information). In contrast, the MC3T3‐E1 cells spread on CG Ti, FG Ti‐T and Ti6Al4V were randomly oriented (Figures S10 and S11, Supporting Information). The cytoskeletons of the cells on FG Ti were also concentrated within small orientation angles of the RD and formed more and longer stress fiber, thereby increasing the cell length and elongation (Figures S9C,D and S12, Supporting Information). Of note, the surface roughness of FG Ti did not differ from that of other materials (Figure S13, Supporting Information). The data suggest that aggregation of the FA complex was influenced by the elongated grains of FG Ti, causing anisotropic arrangement of the actin fibrils and elongation of the cells along the RD. That is, the fibrous‐grained microstructure on FG Ti likely aggregated the FAs, further contributing to the anisotropic arrangement of actin fibrils. Consequently, the cells were elongated along the RD with small orientation angles. Previous researches have demonstrated that the aligned microgrooves which can induce contact induction should be greater than 2 µm in height, 5 µm in width, and 5 µm in spacing.^[^ ^8^ ^]^ And the surface fibrous grains of our material just match these requirements and show average height, width, and spacing of 2.1, 7.2, and 5.8 µm, respectively.

Next, we analyzed the cell motility via the migration trajectory assay (Figure 4C, Supporting Information Videos). The cells on the CG Ti surface showed randomly distributed angles, migrating in any direction from parallel to perpendicular. Some of the cells barely moved from their original position. In contrast, the cells on the FG Ti surface moved parallel to the fibrous grains. Although the average migration velocity of the cells was slightly slower on FG Ti (6.54 µm/10 h) than on CG Ti (8.23 µm/10 h), the average fraction of cell moving time was 50% higher on FG Ti (28.9%) than that on CG Ti (19.4%) (Figure S9E, Supporting Information). For this reason, the travel distance was about 30% further on FG Ti (66.4 µm) than on CG Ti (55.7 µm). The enhanced migration subsequently accelerated the osseointegration processes on FG Ti.^[^ ^20^ ^]^ Unlike the microgrooves formed by varied surface treatments or slip traces introduced by dislocation motion,^[^ ^21^ ^]^ the fibrous grains of FG Ti form a surface‐oriented topological structure that provides excellent contact guidance of osteoblasts (MC3T3‐E1 cells).

To further evaluate the osteointegration ability of the new Ti material, we quantified the expression levels of osteogenic markers in the osteoblasts via quantitative reverse transcription polymerase chain reaction. Among 84 osteogenesis‐related genes, 36 differentially expressed genes presented a 1.2‐fold change or higher (*p* < 0.05). The osteoblast cells expressed more osteogenesis‐related genes on FG Ti than on CG Ti (Figure 4D). The differential genes were then subjected to a gene‐ontology (GO) function analysis. The fibrous surface improved the osteoblast function performance, facilitating bone mineralization, osteoblast differentiation, the signaling pathway of bone morphogenetic proteins, and ossification (Figure 4E).

### 2.5 Hybrid Ti/TiO_2_ Surface with Hydrophilicity for Biomineralization

**Figure 5:**
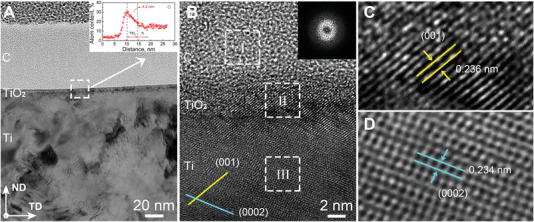
Characterization of anatase TiO_2_ on the FG Ti matrix. A) Typical TEM micrograph showing the microstructure of the surface oxide film on the FG Ti matrix. The inset plots oxygen content versus depth from the surface. B) High‐resolution TEM image showing TiO_2_ anatase formed on the FG Ti matrix. The inset is a selected area diffraction pattern of Region C (white square area I). C) Enlarged image of the white square area II in B, showing a lattice spacing of 0.236 nm for anatase (001). D) Enlarged image of the white square area III in B, showing a lattice spacing of 0.234 nm for Ti(0002).

The highly textured character of the fibrous grains exposed the Ti(0002) planes to the outside of the rod axis (Figure 1B and Figures S1F and S2, Supporting Information). This specific crystal orientation dominated the formation of the crystallographic form of the TiO_2_ layer and affected the following bioactivity in mineralization. We further investigated the surface microstructure of the FG Ti sample, focusing on the surface chemistry of Ti/TiO_2_. High‐resolution TEM and X‐ray photoelectron spectroscopy (XPS) results revealed a sub‐nm TiO_2_ layer on the longitudinal‐sectional plane of FG Ti exposed to air for 30 min (**Figure** **5**A and Figures S14 and S15, Supporting Information).

**Figure 6:**
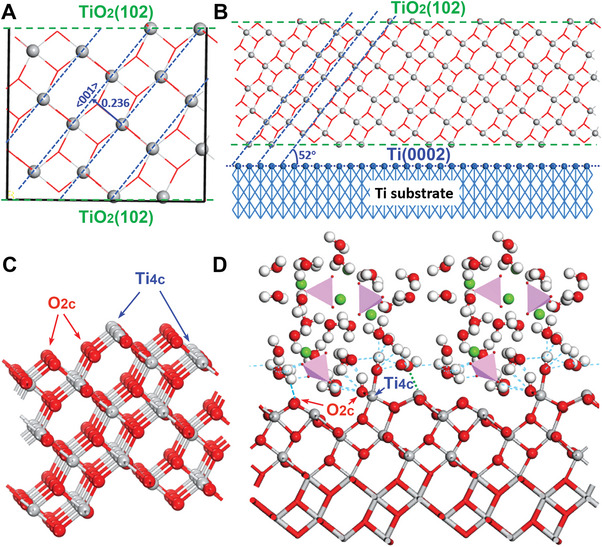
Calculation of anatase TiO_2_ on the FG Ti matrix. A) TiO_2_(102) surface with 〈001〉 orientation and an interlayer distance of 0.236 nm. B) TiO_2_(102)/Ti(0002) heterostructure, featuring with a slope angle of *θ* = 52° between TiO_2_〈001〉 and Ti(0002). C) Unsaturated titanium and oxygen surface atoms are labeled as Ti_4c_ and O_2c_, respectively. D) Ca_10_(PO_4_)_6_(OH)_2_ in water solution loaded on TiO_2_(102), showing partial dissociation of water over Ti_4c_ and a hydrogen network (light blue lines) at the interface. Ti and O are shown as gray and red balls/sticks, respectively. The other constituents are Ca^2+^ (green balls), H (white balls), and PO3‐4 (pink tetrahedrons).

As confirmed in higher resolution images (Figure 5B–D), the Ti substrate was dominated by the low‐indexed Ti(0002) crystalline plane. From the interlayer distance and 〈001〉 orientation, the phase was identified as anatase TiO_2_(102) (Figures 5C,D and 6A). Although (001)‐dominated crystals were successfully synthesized,^[^ ^22^ ^]^ TiO_2_(102) or a TiO_2_(102)/TiO_2_(001) hybrid surface will more likely be exposed in real biological environments than the Ti substrate, as reflected in the hybrid TiO_2_(102)/Ti(0002) heterojunction (**Figure** **6**B). At the atomic scale (Figure 6C), TiO_2_(102) has four‐fold Ti and two‐fold O coordinations, labeled as Ti_4c_ and O_2c_, respectively. Lowly coordinated surface atoms often present a strong water‐dissociation capacity, generating adsorbed H and OH groups.^[^ ^23^ ^]^ Such reactivity is essential for building excellent biocompatibility with a biological system, as it produces hydrogen bonds (HBs) within a watery body. To validate this hypothesis, we introduced an aqueous solution of HA (an important biomineralization agent)^[^ ^10a,b^ ^]^ to the surface and fully optimized the system using density functional theory (Figure 6D). Spontaneous dissociation of water molecules and HBs were observed around Ti_4c_ and O_2c_ over the water/TiO_2_(102) interface. Such an HB‐featured interface offers a friendly environment for HA (Ca^2+^: green balls; PO3‐4: pink tetrahedrons) interaction with OH groups (see Figure 6D). The simulation results confirm the excellent hydrophilicity of the anatase TiO_2_(102)‐dominated surfaces, as further supported by the measured water contact angles (see Figure S16, Supporting Information). The water droplets on FG Ti were shuttle‐shaped, being extended in the RD and resembling the cells spread over FG Ti. This phenomenon might drive the direction of the biomineralization process.

Similar simulations were conducted on dominant surfaces anatase TiO_2_(101) and the TiO_2_(102)/TiO_2_(001) hybrid. The results are presented in Figure S17 (Supporting Information). The optimized geometries showed favorable water dissociation over TiO_2_(102) and TiO_2_(102)/TiO_2_(001), but not over TiO_2_(101), consistent with previous reports.^[^ ^12c^ ^]^ Accordingly, a radical distribution function (RDF) was plotted and integrated over the typical HB bonding distance (1.5–3.0 Å, see Figure S17, Supporting Information). On the TiO_2_(102) and TiO_2_(102)/TiO_2_(001) surfaces, O—H bonding associated with water dissociation effectively promoted interfacial infiltration, as evidenced by RDF integration between the TiO_2_ matrix and HA/water body. Consistent with this calculation, early studies reported that the hydrophilic surfaces are essential for biocompatibility and cell growth.^[^ ^7^, ^24^ ^]^ Therefore, they likely explain the distinguished osteogenesis performance of FG Ti. And the TiO_2_ configuration determined from crystallographic properties of the titanium matrix plays a crucial part in biomineralization. Among three different crystallographic forms of TiO_2_, anatase best accelerates the biomineralization^[^ ^25^ ^]^ and cell proliferation^[^ ^26^ ^]^ because its lattice well matches that of HA and its *ζ*‐potential is lowered by the larger number of surface hydroxyl groups. To confirm the calculation results, we finally conducted a biomineralization‐mimicking experiment in a simulated body fluid. Bone‐like apatite formed at 48 h and thickened at 96 h. Such vigorous growth was not observed on CG Ti (Figure S18, Supporting Information). X‐ray diffraction (XRD) analysis further demonstrated that the formed layer on FG Ti mainly consists of HA (PDF#74‐0565), showing typical peaks at 15.9^o^, 16.1^o^, and 16.5° corresponding to the crystal planes of (211), (112), and (300) of HA. While HA peaks were inconspicuous of CG Ti, which meant the quantity and crystallinity of formed HA on CG Ti was inferior to that on FG Ti (Figure S19, Supporting Information).

Thus, high‐resolution TEM revealed subnanometer anatase on titanium with (0002) as the main crystallographic plane. The anatase thin film strongly dissociates water, generating adsorbed H and OH groups and creating a favorably hydrophilic surface for biomineralization. As confirmed in the computational simulation, the predominant basal Ti(0002) crystal plane of FG Ti induces the formation of hydrophilic anatase TiO_2_ layers that further benefit the following biomineralization.

## 3 Conclusion

In summary, we report a hierarchical FG pure Ti that combines exceptional mechanical properties and outstanding osseointegration characteristics, mimicking those of natural bone. This biomimetic microstructure with a specific crystallographic texture not only enhances tensile strength but also facilitates the oriented adhesion and migration of bone cells. In particular, the fiber texture exposes the low‐indexed Ti(0002) crystalline plane to air, allowing the formation of hydrophilic TiO_2_ film with crystallographic anatase. This film substantially accelerates the osseointegration process. Importantly, the ultrafine fiber‐like microstructure can be fabricated by conventional extrusion and a rotary swaging process, which can be economically up‐scaled for industrial production.

## 4 Experimental Section

### Materials

Commercial pure titanium (ASTM Grade 2, Ti) and titanium alloy (Ti6Al4V) rods were made by the Baoji titanium industry company (China). Nitric acid (HNO_3_, 68%, w/w), hydrofluoric acid (HF, ≥ 40%, w/w), and saline were bought from Sichuan Kelun Pharmaceutical CO., LTD. (China). Enrofloxacin (C_19_H_22_FN_3_O_3_) was bought from Adams (China). Pentobarbital sodium (C_11_H_17_O_3_N_2_Na) was obtained from Ailu Biological Tech Co. Ltd. (China). Povidone‐iodine was from Jinshan Pharmaceutical Company (China). Calcein (C_30_H_26_N_2_O_13_), toluidine blue, and Giemsa stain solution were from Solarbio (China). Aluminum oxide (Al_2_O_3_) particles of 40 grit (0.4 mm in diameter) for sandblasting were obtained from Bioactive Material Co. Ltd. (BAM, China). Vinculin antibody, basic fuchsin, and rhodamine‐conjugated phalloidin were from Sigma (USA). FITC conjugated goat‐anti‐rabbit secondary antibody, Trizol reagent, 4', 6‐diamidino‐2‐phenylindole (DAPI), and 5‐chloromethylfluorescein diacetate (CMFDA) were from Thermo (USA). SuperScript III Reverse Transcriptase was from Invitrogen (USA). The PCR array of mouse osteogenesis‐related genes (RT^2^ Profiler PCR Array Mouse Osteogenesis, PAMM‐026Z) was purchased from QIAGEN.

### Fabrication of FG Ti

The FG Ti samples were fabricated by means of hot extrusion and cold rotary swaging, together with annealing treatment. The starting commercial pure Ti rods with a dimension of Ø100 mm × 120 mm were vacuum‐annealed at 1073 K for 2 h first to obtain a homogenous coarse‐grained (CG) microstructure with a mean grain size of 36 ± 8 µm. Thereafter, the annealed thick Ti rods were extruded at 450 °C to thin rods with a diameter of Ø20 mm. Finally, the as‐extruded thin Ti rods were subjected to rotary swaging at room temperature, producing fine rods with a diameter of Ø5 mm and a length of 1000 mm. The accumulative strains for the processes of hot extrusion and cold swaging were 3.22 and 2.77, respectively, evaluated by *φ* = ln(*A*_0_/*A*), where *A*_0_ and *A* are the initial and final cross‐sectional areas. No cracks were observed on the surface of the Ti rods during the processes of hot extrusion and cold swaging. After deformation processing, the fine Ti rods were further annealed (protected by N_2_ gas) at 475 °C for 6 min to form the FG microstructure.

### Uniaxial Tensile Test

For the tensile test, flat dog‐bone‐shaped tensile specimens with a gauge length of 12 mm and a width of 1.8 mm were cut from the rods along the longitudinal direction by electrical discharge machining. Before the tensile test, all specimens were mechanically and electrochemically polished to minimize surface roughness. Quasi‐static uniaxial tension, a strain rate of 5 × 10^−4^ s^−1^ at ambient temperature, was carried out using a testing machine (BOSE ElectroForce 3230 DMA, Shimadzu, Tokyo, Japan). In the tensile tests, an extensometer with a gauge length of 10 mm was used to monitor the strain. Five specimens were tested to verify the reproducibility.

### Microstructural Characterization

*TEM observations*: The microstructures of FG Ti were examined by a JEM 2100F FEG TEM operating at 200 kV. Thin foils were sliced from the FG Ti rods along the transversal and longitudinal sections, followed by mechanically polishing to a final thickness of about 80 µm. The final thinning was performed by twin‐jet polishing using a solution of perchloric acid (5%), butanol (35%), and methanol (60%). High‐resolution TEM (HRTEM) foils were prepared with an FEI Scios DualBeam focused ion beam (FIB). The milling voltage/current was 2 kV/34 pA. Before the FIB process, bulk samples were mechanically polished using standard metallographic techniques and then electronically polished to remove the strained surface layer. To examine the crystal information of the oxide film on the FG Ti sample surface, the oxide film formed by natural oxidation for 30 min was protected by covering a carbon film. Thereafter, TEM foils were elaborately extracted using the FIB lift‐out technique and then examined on an FEI Tecnai G2 T20 microscope at 200 kV.

*EBSD observations*: EBSD analysis was performed on scanning electron microscopy (SEM, JEOL JSM‐7001F) equipped with a DigiView camera and Channel 5 software, to examine the crystallographic texture and microstructure of FG Ti samples. The scanning step size and accelerate voltage were 80 nm and 15 kV, respectively. The EBSD samples, in a dimension of Ø8 mm × 10 mm, were cut from the FG Ti rods along the transversal and longitudinal sections. Mechanical and electrolytic polishing were sequentially conducted to prepare a mirror‐like surface.

*XRD measurements*: The texture of CG Ti, FG Ti‐T, and FG Ti‐L samples were further confirmed by XRD (X'Pert PRO, Netherlands) with a scanning step size of 0.04° within 20° to 60° at 40 V (generator voltage) and 40 A (tube current). The preparation of XRD samples was the same as that for EBSD observation.

*XPS measurements*: The elemental composition and valence state of the elements were analyzed by XPS. All specimens were electropolished and exposed to air for 30 min. The specimens were then rinsed with deionized water and dried with highly purified nitrogen before XPS analysis. XPS analysis was performed on PHI 5000 VersaProbe III instrument (monochromatic Al K (*h* = 1486.6 eV)). The vacuum of the chamber was kept at 5 × 10^−10^ Torr. The calibration of the XPS spectra was done by C 1s peak position (284.5 eV).

### Preparation and Characterization of Titanium Screw Implant for In Vivo Studies

*Screws implant preparation*: Rods of FG Ti, CG Ti, and Ti6Al4V in diameter of 8 mm were machined into slotted screws along the longitudinal axis according to the computer‐aided design (CAD) sketch, consisting of a cylindrical head and thread with taper. The 3 mm diameter cylindrical head was designed to avoid the whole screw falling into the marrow cavity. The slot through the middle of the screw head was for subsequent torque tests. Thereafter, all screws were treated with sandblasting and acid etching for roughness enhancement to increase primary implant stability with improved mechanical fixation. They were sandblasted with 40 grit (0.4 mm in diameter) aluminum oxide particles vertically from a nozzle under 0.3 MPa pressure for 15 s. After that, samples were ultrasonically cleaned in deionized water for 30 min, followed by acid etching for 22–25 s at 25 °C in a solution composed of HNO_3_ (8%), HF (5.5%), and deionized water (86.5%) in a polytetrafluoroethylene beaker. Acid etching aimed to remove the extra aluminum oxide particles and reduce the surface roughness. And then, the screws underwent a five‐time ultrasonic clean in deionized water, ethanol dehydration, and drying successively at 110–120 °C for 30 min. After all treatments, the surface microstructure of the crew was observed by SEM (Hitachi S‐4800, Japan). While samples for in vivo implantation were sterilized by gamma irradiation at 25 kGy overnight.

*Surgical procedures*: All animals were kept in a pathogen‐free environment with ad libitum feeding. The procedures for the care and use of animals were approved by the Ethics Committee of the Huaxi Animal Experiment Center of Sichuan University with Ethics Record number 20211196A, and all applicable institutional and governmental regulations concerning the ethical use of animals were followed. *New Zealand white* rabbits of 2.2–2.5 kg were randomly assigned into three groups for FG Ti, CG Ti, and Ti6Al4V implant placement. Each group was consisted of 12 rabbits for Ti screw implantation. After anesthesia by pentobarbital sodium (40 mg kg^−1^) via a marginal ear vein injection, the limbs of rabbits were immobilized to remove the hair and Povidone‐iodine was used to sterilize for following surgery operation. A longitudinal incision was made on the medial surface of the femur, and a 2.0 mm diameter drill was used to drill a hole into the exposing femur with a speed of 800–1200 rounds per minute. During the process, the temperature was continuously cooled with normal saline rinsing. Two Ti screws were implanted into the flat surface of the distal femur metaphysis in per leg. To reduce postoperative pain, all rabbits were given a Finadyne injection for 2 days after the operation. At the same time, enrofloxacin was intramuscularly injected at 10 mg kg^−1^ to reduce the risk of postoperative infection.

*Torque tests*: The rabbits were sacrificed with the intravenous injection of excessive amounts of pentobarbital sodium (80 mg kg^−1^) at 3, 7, and 12 weeks after implantation. The femurs were harvested and fixed on a torque sensor (A‐BF‐10, China) with a measurement range of 0.015–1 N m and a sampling frequency of 30 s^−1^. A screwdriver was used to unscrew the screw anticlockwise, with a measurement speed of 3^o^ s^−1^ for torque record. During the test, the axis of the implant corresponded exactly with the axis of the torque sensor. The torque‐angle curve was recorded by equipment auxiliary software.

*Micro‐CT evaluation*: Femurs of each group with screws were scanned by a professional Micro‐CT system (SCANO vivaCT‐80, Switzerland) with a resolution of 6.5 µm at 70 kV, 114 µA, and 700 ms integration time. The new bone and trabecular microarchitecture parameters were determined within the scope of the hole drilled before implantation around implants (2 mm in diameter). The 2D images obtained from continuous slices including all new bone were selected for 3D reconstruction by equipment auxiliary software. A global threshold was utilized to segment the newly formed bone from each implant. After thresholding, the newly formed bone around the implant was quantitatively determined by normalized bone volume (BV), the ratio of BV to total volume (BV/TV), bone surface area (BS), the ratio of BS to total volume (BS/TV), bone trabecula number (Tb. N) and trabecular separation (Tb. Sp) of the defect area.

*Histological evaluation*: Samples harvested from animals were fixed in 10% formalin, dehydrated with series ethanol solutions (70%, 80%, 90%, 95%, and 100% × 2), and subsequently embedded in poly(methylmethacrylate) (Cool‐Set‐A, Aorigin, China). Sections (10–20 µm of thickness) were cut with a diamond histological saw (SAT‐001, Aorigin, China), and stained with 1% toluidine blue or 0.3% basic fuchsin for 1 min for histological observation (Leica DM 1000). Tissue sections in the early period of osteogenesis (3 weeks) were stained with toluidine blue, and those in the later period of osteogenesis (12 weeks) were stained with basic fuchsin. Rabbits were intraperitoneally injected with calcein staining solution (10 mg kg^−1^) at 3 weeks. The femurs were harvested 3 days after injection and subjected to the hard tissue sections preparation. The fluorescence of calcein was observed by the laser scanning confocal microscope (Zeiss LSM 880) with an excitation wavelength of 405 nm.

### In Vitro Studies on Biomineralization and Its Effect on Osteo‐Related Cells

Titanium disks preparation, surface modification, and characterization: Metal disks of FG Ti, FG Ti‐T, CG Ti, and Ti6Al4V were prepared by electrical discharge machining with a size of 8 × 8 × 1.5 mm (length × width × height) from rods in diameter of 8 mm. Longitudinal section of FG Ti was termed as FG Ti. After ultrasonic cleaning with acetone, ethanol, and deionized water successively, all metal disks were polished with 200–2000 grit carborundum papers, cleaned again, and dried in an air oven at 50 °C for 2 h. Acid etching was performed as described above, and the treated Ti disks were subjected to the following in vitro studies, as they showed a more intact fibrous and flat surface than the samples with sandblasting and acid etching treatment. SEM (Hitachi S‐4800) was used to determine the surface microstructure of titanium screws (FG Ti, CG Ti, and Ti6Al4V) with different surface treatments. Surface roughness was characterized by atom force microscope (AFM, Asylum Research, MFP3D‐BIO) within the area of 90 × 90 µm. The surface hydrophilicity was examined by a water contact angle meter (Attension Theta Lite). Briefly, 5 µL of water was dropped onto the FG Ti titanium disks and observed vertically and parallelly to the rolling direction. While the contact angles of water on CG Ti and Ti6Al4V served as control. Electrolytic polishing was performed in electrolyte composed of HF (10%), H_2_O (20%), and H_2_O_2_ (70%). Sample was used as the anode, while a platinum sheet with a size of 20 × 20 × 0.2 mm was used as the cathode. They were parallel to each other with a separation distance of 50 mm, and suffered to 30 V by a direct‐current power (Keithley 6221) for 30 s.

*In vitro biomineralization*: Disks of FG Ti and CG Ti were immersed in 500 mL simulated body fluid (SBF) at 37 °C for 24, 48, and 96 h after acid etching. 1 L SBF was composed of NaCl (8 g), CaCl_2_ (0.3 g), NaHCO_3_ (0.355 g), NaH_2_PO_4_·12H_2_O (0.358 g), KCl (0.225 g), MgCl_2_·6H_2_O (0.311 g), NaSO_4_ (0.072 g), and 1 L distilled water with the pH of 7.4. The solution was re‐changed at 24 h intervals.

*Orientation analysis of cell adhesion*: Mouse osteoblast precursor cells MC3T3‐E1 were routinely cultured by the complete *α*‐MEM medium in a humidified cell culture incubator containing 5% carbon dioxide at 37 °C. Cells were seeded onto the Ti disk (8 × 8 × 1.5 mm) at a density of 5 × 10^4^ mL^−1^ in a 24‐well plate. For the adhesion orientation analysis, cells were fixed by methanol for 10 min, stained with 5% Giemsa stain solution after 36 h culture, and observed by a metallographic microscope. The orientation was analyzed via the image processing software Image J.

*Focal adhesion and cytoskeleton organization*: The MC3T3‐E1 cells were cultured and seeded as described above. After removing the culture medium, cells were in turn fixed by 4% paraformaldehyde, permeabilized by 0.1% Triton X‐100, and blocked by 5% bovine serum albumin. And then, they were thoroughly washed, stained orderly with the vinculin antibody (1:500) overnight at 4 °C, the FITC‐conjugated goat‐anti‐rabbit secondary antibody (1:5000) for 1 h, rhodamine‐conjugated phalloidin (1:250) for 1 h, and DAPI (1:5000) for 5 min at room temperature. Finally, cells were washed and observed by a confocal microscope (Zeiss LSM 880).

*Living cell movement imaging and analysis*: The MC3T3‐E1 cells were cultured and seeded as described above. A living cell tracker CMFDA (Thermo) was then added at a final concentration of 0.5 × 10^−6^ m. After 30 min incubation and labeling, cells were observed and monitored by the live cell imaging system (DMI6000B, Leica) for 12 h at 5 min intervals. The cell motion was tracked using a Track Particle module equipped with the same confocal laser scanning microscope (CLSM), which can analyze the trajectory automatically. The movement trajectories of the cells were determined and analyzed using MATLAB software.

*Osteogenesis quantitative polymerase chain reaction (q‐PCR) array*: The MC3T3‐E1 cells were cultured and seeded as described above. The culture medium was replaced every other day. After 7 days of osteogenesis induction with OS medium (0.1 × 10^−6^ m dexamethasone, 50 × 10^−6^ m ascorbic acid, and 10 × 10^−3^ m *β*‐sodium glycerophosphate), at least 1 × 10^6^ cells in each group were collected and lysed by Trizol reagent. Total RNA was extracted by chloroform, isopropanol, and 70% ethanol followed by transcription to cDNA by SuperScript III Reverse Transcriptase. The PCR array of mouse osteogenesis‐related genes was performed in an ABI QuantStudio 3 Real‐Time PCR system.

### Models of Computational Methods

*Ti/TiO_2_ heterojunction*: The titanium matrix was simulated by its surface Ti(0002), cleaved from hcp titanium (*a* = *b* = 2.95 Å, *c* = 4.68 Å, *γ* = 120°). To match with anatase TiO_2_(102) (*a* = 12.13 Å, *b* = 3.78 Å, *γ* = 90°), the lattice of hexagon Ti(0002) had been refined with new lattice vectors (*a’* = 2.95 Å, *b’* = 5.11 Å, *γ* = 90°), following which the heterojunction Ti/TiO_2_ had been fabricated using the supercells of Ti(0001)‐(4×3) and TiO_2_(102)‐(1×4). Under this scheme, a heterojunction with *a* = 11. 96 Å and *b* = 15.21 Å had been built, with the maximum lattice mismatch of 1.4% and 0.8% along the *a* and *b* direction, respectively. Such a small mismatch supported the observed heterojunction Ti/TiO_2_ shown in Figure 6A,B.

*TiO_2_ surfaces*: To simulate the water/TiO_2_ interface, ideal TiO_2_(102) and TiO_2_(101) had been modeled by the supercells (102)‐(1×2) and (101)‐(2×2), with dimension sizes of 12.13 Å × 7.55 Å and 10.88 Å × 7.55 Å, respectively. These slabs contained three to five layers of TiO_2_, with two to three bottom layers fixed during the optimization. Defective TiO_2_(102) had been further investigated by introducing TiO_2_(102)/TiO_2_(001) steps, by removing several rows of Ti/O atoms, under which V‐shape defects featured with TiO_2_(001) surfaces (dominated by five‐coordinated Ti and two‐coordinated O).

*HA model*: For the perfect HA crystal, its formula is Ca_5_(PO_4_)_3_OH. To simulate HA aqueous solution, single Ca_5_(PO_4_)_3_OH molecule had been introduced to the water solution (28 water molecules). The initial configuration had been fully optimized and relaxed using force‐field molecular dynamics (COMPASS force field, as described below).^[^ ^27^ ^]^

*Water model*: To simulate the water solution, 28 water molecules were introduced to TiO_2_ surfaces. Water clusters were relaxed using force field molecular dynamics. Specifically, water molecules close to TiO_2_ surfaces were tested with molecular and dissociative adsorption geometries, with energetically favorable geometries for following full optimization of HA/water/TiO_2_ structures.

### Force Field Molecular Dynamics

In primary optimizations, the COMPASS force field was employed to relax HA and water clusters using the program Forcite, as embedded in Materials Studio. To relax the water body, molecular dynamics was carried out with time step Δ*t* = 1 fs with a full simulation of 500 ps. Initial velocities were randomly set based on simulation temperature *T* = 298 K, with NVE ensemble. Under this setting, the maximum temperature fluctuation was less than 100 K. Final geometries from molecular dynamics had been used for advanced optimization under the scheme of density functional theory.

### Density Functional Theory Calculations

In this case, density functional theory calculations were performed using the CASTEP.^[^ ^28^ ^]^ Specifically, the Perdew–Burke–Ernzerhof exchange‐correlation functional within the generalized gradient approximation was employed to describe the exchange‐correlation energy, together with ultrasoft pseudopotentials.^[^ ^29^ ^]^ The energy cutoff for the plane wave basis expansion was set to 450 eV. The convergence criteria for total energy and force on each atom were set as 10^−4^ eV and 0.05 eV Å^−1^, respectively. The slab model was constructed with a vacuum layer of 15 Å in the *z*‐direction to avoid the interaction between layers. The sampling in the Brillouin zone for all surface slabs was set with 1×1×1 by the Monkhorst‐Pack method.^[^ ^30^ ^]^ The van der Waals interaction was considered using the DFT‐D dispersion scheme.^[^ ^31^ ^]^

## Conflict of Interest

The authors declare no conflict of interest.

## Author Contributions

R.W. and M.W. contributed equally to this work. Conceptualization: Y.N., C.H. Methodology: M.Y., J. L. Investigation: R.W., M.W., R.J., Y.W. Simulation: Q.L., C.S. Supervision: Y.N., C.H., A.G.M., X.Z. Writing—original draft: R.W., M.W., R.J., Y.W., C.S., Y.N., C.H. Writing—review & editing: Y.N., K.Z., C.H.

## Supporting information

Supporting Information

Supplemental Video 1

Supplemental Video 2

Supplemental Video 3

## Data Availability

The data that support the findings of this study are available from the corresponding author upon reasonable request.
