# Supplementary material for: High Strength Titanium with Fibrous Grain for Advanced Bone Regeneration
Source: Adv Sci (Weinh). 2023 Apr 7;10(16):2207698. doi: 10.1002/advs.202207698 (PMC10238201; doi:10.1002/advs.202207698)
Supplement: Supplementary file 1 — Supporting Information [file ADVS-10-2207698-s002.pdf]

ADVANCED SCIENCE Open Access

Supporting Information

for *Adv. Sci.*, DOI 10.1002/advs.202207698

High Strength Titanium with Fibrous Grain for Advanced Bone Regeneration

Ruohan Wang, Mingsai Wang, Rongrong Jin, Yanfei Wang, Min Yi, Qinye Li, Juan Li, Kai Zhang, Chenghua Sun, Yu Nie*, Chongxiang Huang*, Antonios G. Mikos and Xingdong Zhang*

Supporting Information

High-Strength Bioinspired Fibrous-Grained Titanium for Advanced Bone Regeneration

Ruohan Wang,[1,]† Mingsai Wang,[2,]† Rongrong Jin,[1,] Yanfei Wang,[2,] Min Yi,[3] Qinye Li,[4] Juan Li,[5] Kai Zhang,[1] Chenghua Sun,[4] Yu Nie,[1]* Chongxiang Huang,[1,2]* Antonios G. Mikos,[6] and Xingdong Zhang[1]*

1 National Engineering Research Centre for Biomaterials/College of Biomedical Engineering, Sichuan University, Chengdu 610065, China.

2 School of Aeronautics and Astronautics, Sichuan University, Chengdu 610065, China.

3 Department of Orthopedics, Orthopedic Research Institute, West China Hospital, Sichuan University, Chengdu 610041, China.

4 Department of Chemistry and Biotechnology, Centre for Translational Atomaterials, Swinburne University of Technology, Hawthorn, VIC 3122, Australia.

5 State Key Laboratory of Oral Diseases, West China School of Stomatology, West China Hospital of Stomatology, Sichuan University, Chengdu 610041, China

6 Departments of Bioengineering, Chemical and Biomolecular Engineering, Rice University, Houston, TX 77251, USA.

***Corresponding author:**

chxhuang@scu.edu.cn; nie_yu@scu.edu.cn; chenghuasun@swin.edu.au

† These authors contributed equally to this work

Supporting information includes:

- Supplementary Text
- Figure S1 to S11
- Table S1 to S2
- Movie S1 to S11
- Acronyms

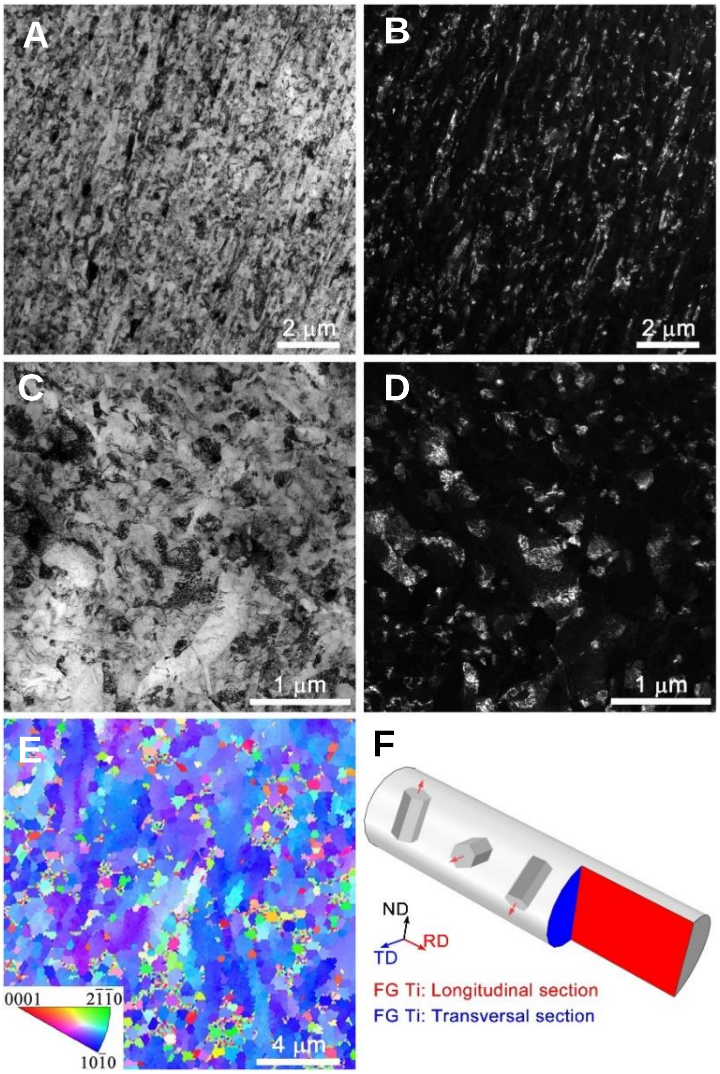

Figure S1. TEM and EBSD microstructures of FG Ti samples. A) and B) Typical bright-field and corresponding dark-field TEM micrographs showing the longitude-sectional (TD-RD plane in Figure 1A) microstructures taken from FG Ti-L sample. C) and D) Typical bright-field and corresponding dark-field TEM micrographs showing the cross-sectional (TD-ND plane in Figure 1A) microstructures taken from FG Ti-T sample. E) Typical EBSD inverse pole image showing the highly orientated microstructure on the TD-ND plane. F) A schematic illustration of the Ti rod and the definition of the sample coordinate system. RD, TD, and ND represent the rolling, transverse and normal directions, respectively. FG Ti-L and FG Ti-T represent the samples sectioned along the longitudinal direction (parallel to the rolling direction) and transversal direction (perpendicular to the rolling direction).

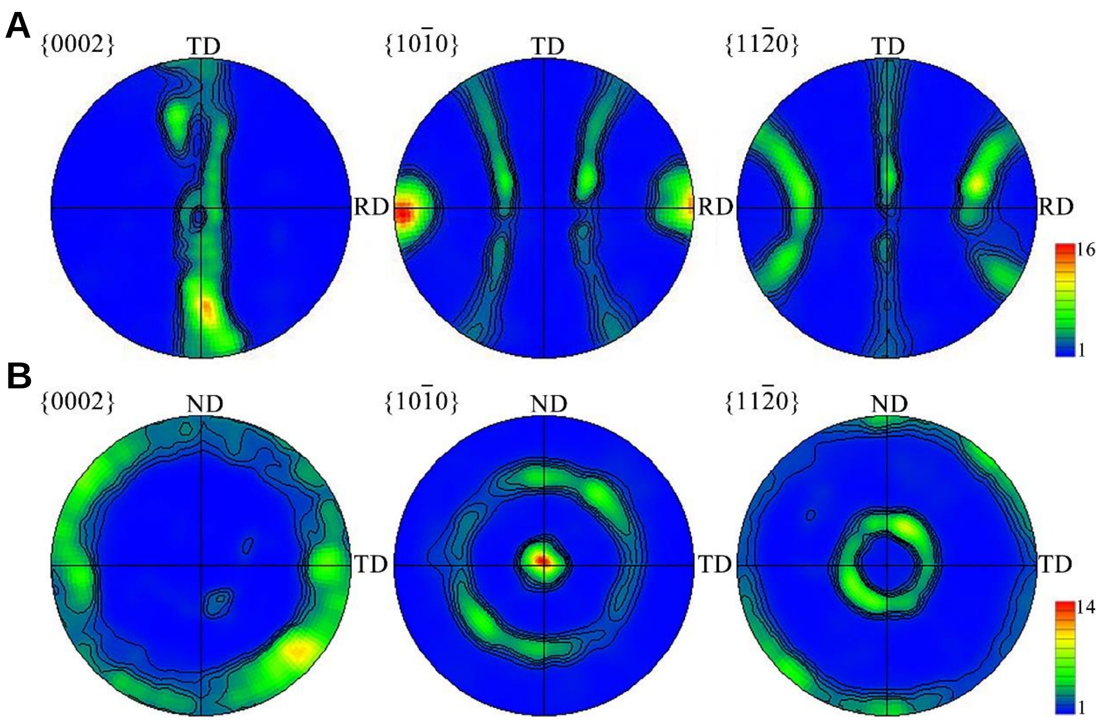

Figure S2. The (0002), (10$\bar{1}$0) and (11$\bar{2}$0) pole figures based on electron back scattered diffraction measurements showing the strong fibrous texture of FG Ti rod. A) FG Ti-L sample. B) FG Ti-T sample.

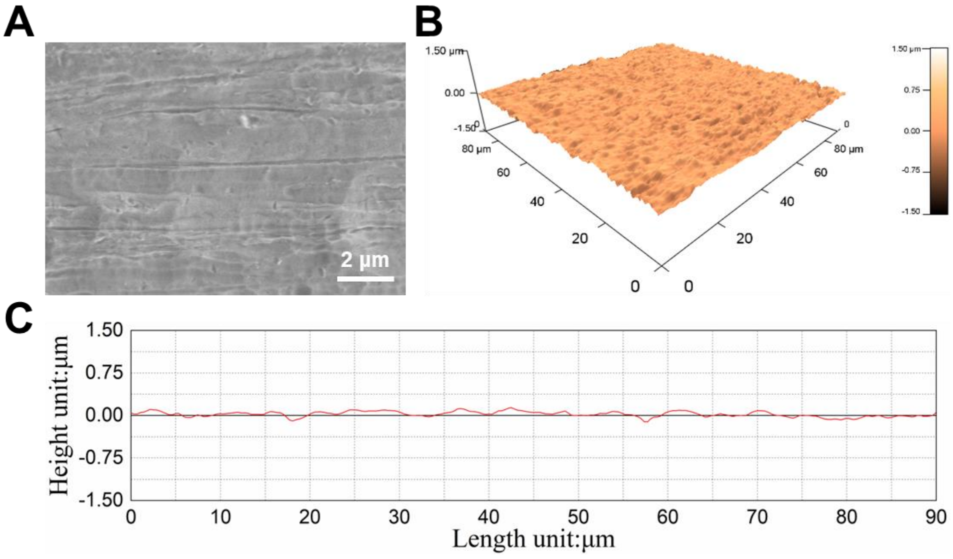

Figure S3. Surface characterization of FG Ti after electrolytic polishing by A) SEM, B) and C) AFM.

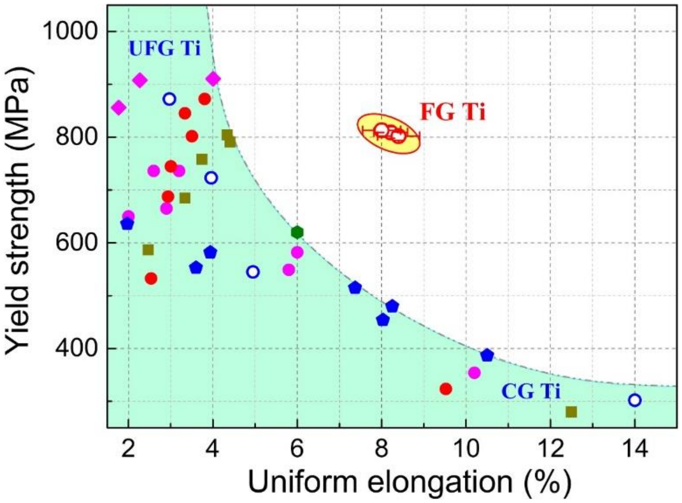

Figure S4. Yield strength versus uniform elongation, compared with the mechanical properties of CG Ti, UFG Ti, and FG Ti.

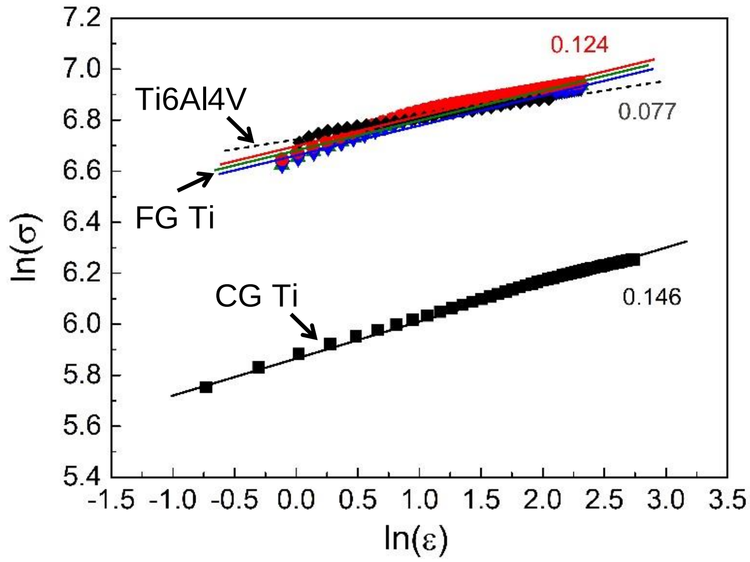

Figure S5. Strain-hardening rates determined from the slop of ln(σ)-ln(ε) plot for FG Ti, CG Ti, and Ti6Al4V samples.

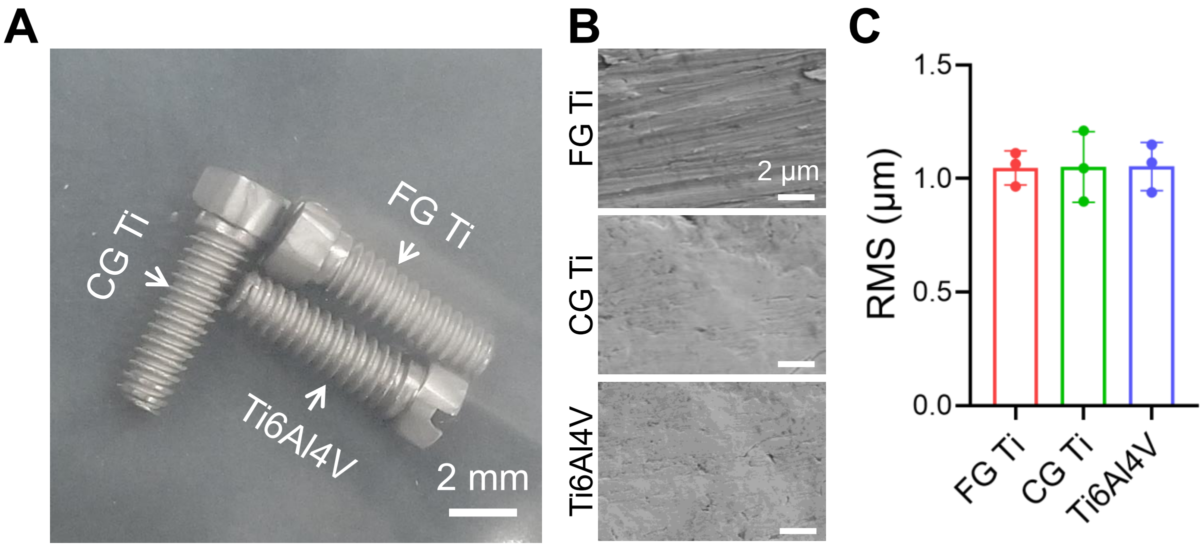

Figure S6. A) and B) Surface morphology before sandblasting and acid etching. C) Surface roughness root mean square (RMS) of varied screws of FG Ti, CG Ti, and Ti6Al4V after sandblasting and acid etching.

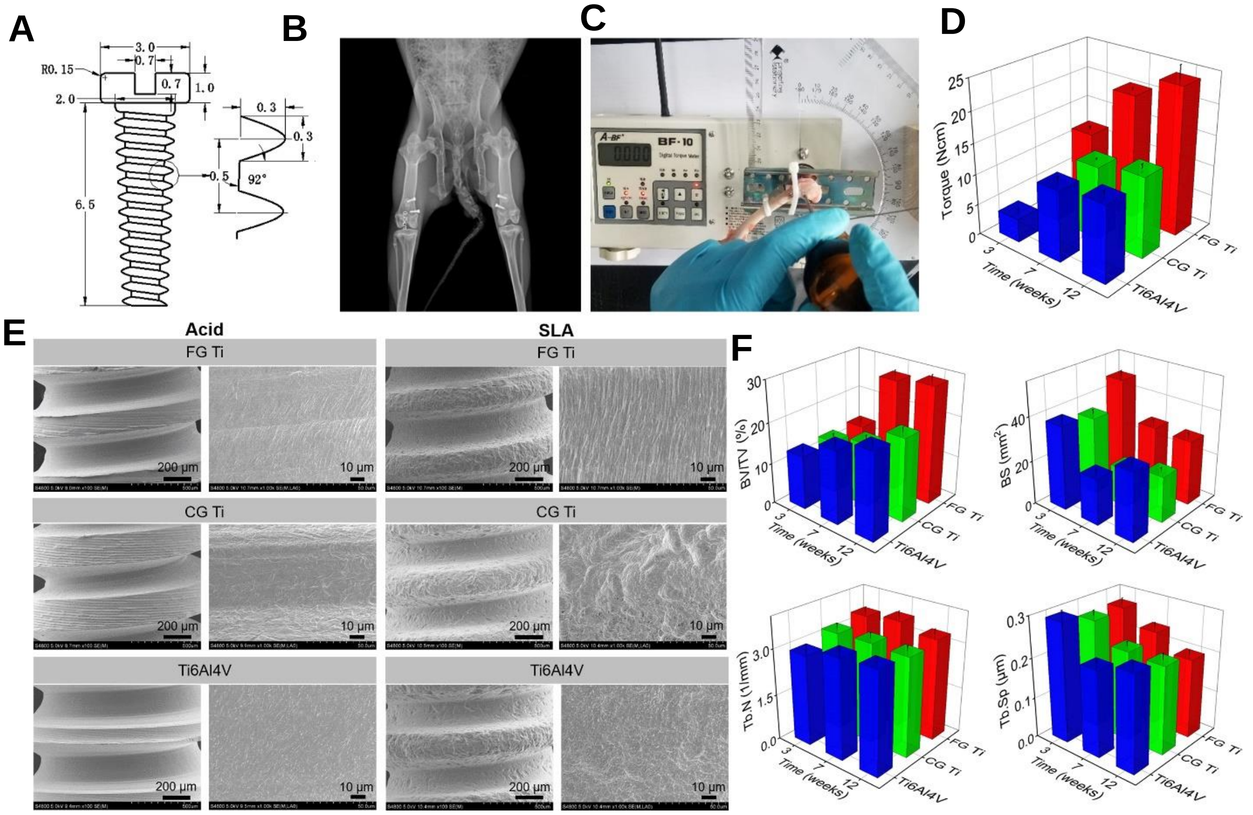

Figure S7. Morphology, animal surgery operation, torque test, and Micro-CT analysis of implants. A) Computer-aided design image of the implant by AutoCAD software. B) Digital radiography X-ray image of the rabbit after implantation. Two holes with a diameter of 2 mm and 10 mm from each other were drilled into the marrow cavity for implantation. C) Torque test demonstration. The measurement of the torque was at the speed of 3 °/s. D) Maximum torque of varied implants at 3, 7, 12 weeks after implantation. E) Surface morphology observation of various implants composed of FG Ti, CG Ti and Ti6Al4V with different surface treatments by SEM. F) Relative parameters of Micro-CT analysis: Bone volume to total volume (BV/TV), bone surface (BS) area, bone trabecula number (Tb. N) and trabecular separation (Tb. Sp) of newly formed bone at 3, 7, and 12 weeks after implantation. All parameters were calculated from the 3D reconstruction model by Micro-CT auxiliary software with a resolution of 6.5 μm.

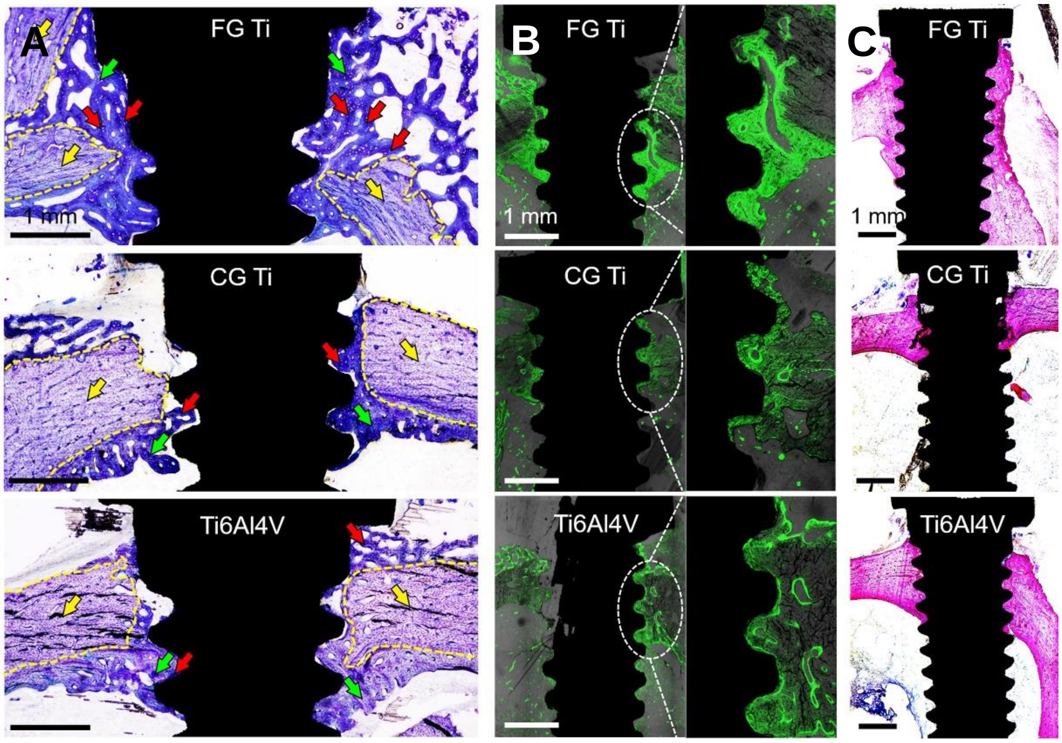

Figure S8. Osseointegration effect of FG Ti, CG Ti, and Ti6Al4V at 3 and 12 weeks after implantation. A) Optical microscope images of bone tissue stained with toluidine blue around the implants at 3 weeks after implantation. black bar = 1 mm. B) The newly formed bone around the implants at 3 weeks after implantation. The tissue sections were observed by confocal laser scanning microscope (CLSM) with 488 nm excitation. Calcein solution (1%, w/w) was intraperitoneally injected (1 ml/kg) for new bone staining 3 d before execution. white bar = 1 mm. C) The tissue sections of various implants at 12 weeks after implantation with newly formed bone stained by basic fuchsin. black bar = 1 mm.

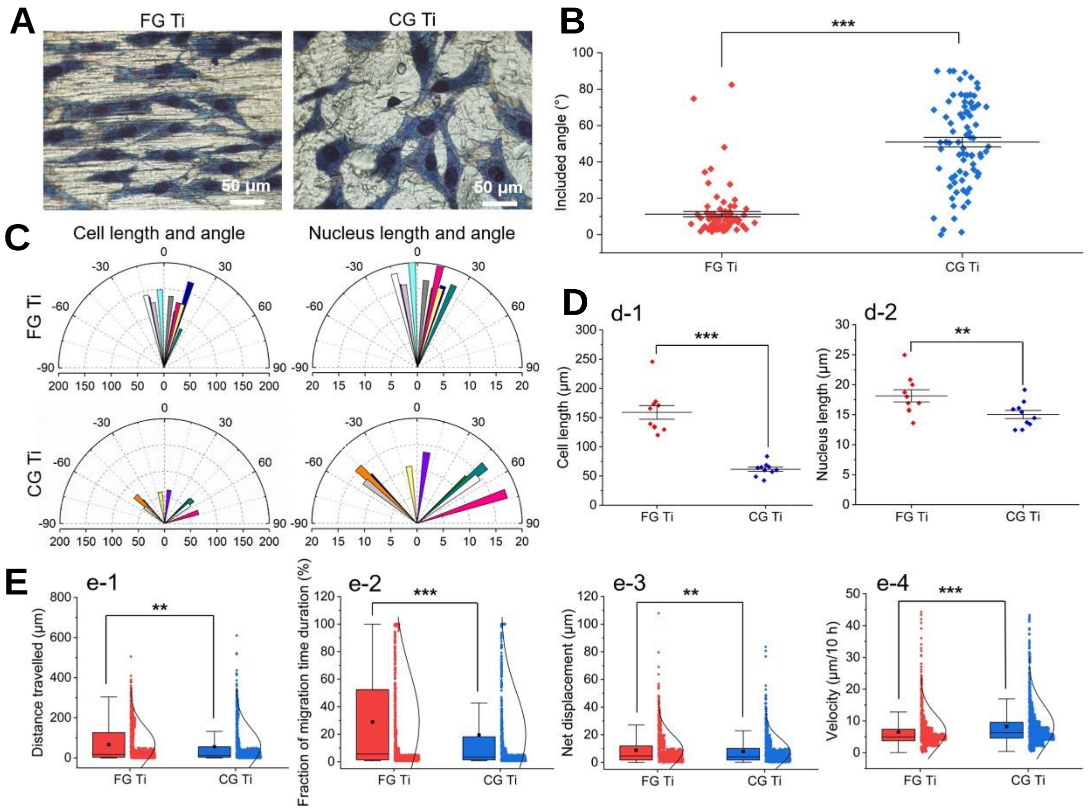

Figure S9. The behaviors of MC3T3-E1 cells on FG Ti and CG Ti after acid etching. A) Cell alignment and morphology observation by metallographic microscope on FG Ti and CG Ti after 36 h spreading. white bar = 50 μm. B) Distribution of included angles (°) between cytoskeletons and RD on FG Ti or arbitrary direction on CG Ti. n = 80. C) Polar plots of cell length (μm) or nuclei length (μm) and angles (°) between cytoskeletons and RD on FG Ti or arbitrary direction on CG Ti. 10 typical MC3T3-E1 osteoblasts on FG Ti and CG Ti from CLSM images of Figure 3B were selected for analysis. D) Average cell length and nuclei length of MC3T3-E1 cells after 36 h spreading on FG Ti and CG Ti. E) Relative parameters of MC3T3-E1 osteoblasts migration on FG Ti and CG Ti. Migration distance of MC3T3-E1 osteoblasts on FG Ti and CG Ti during 12 h after 36 h spreading. Fraction of migration time duration, net displacement, and velocity of MC3T3-E1 osteoblasts migration on FG Ti and CG Ti during 12 h. $^{**}P < 0.01$, $^{***}P < 0.001$.

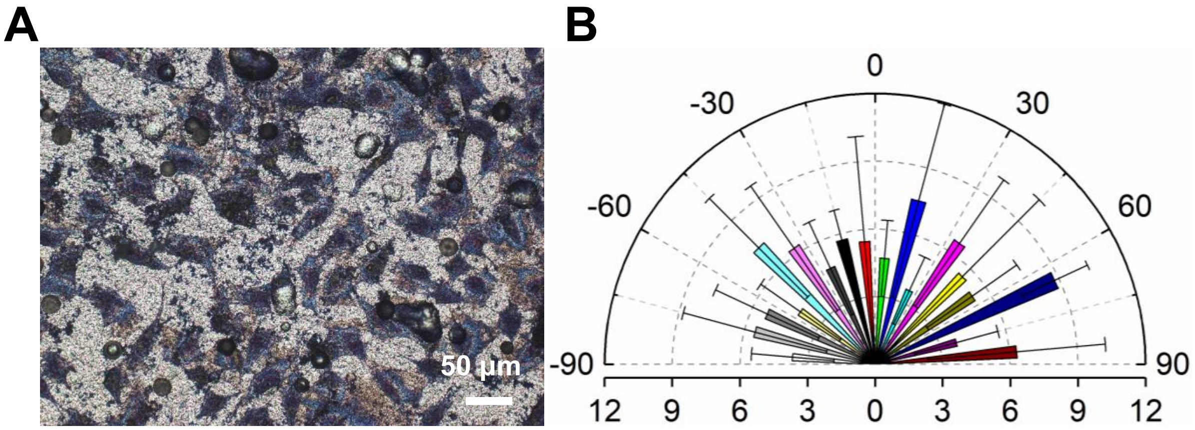

Figure S10. Cell spreading on FG Ti-T. A) Cell alignment and morphology observation by metallographic microscope on FG Ti-T after 36 h spreading. B) Distribution of included angles (°) between cytoskeletons and arbitrary direction on FG Ti-T. n = 80.

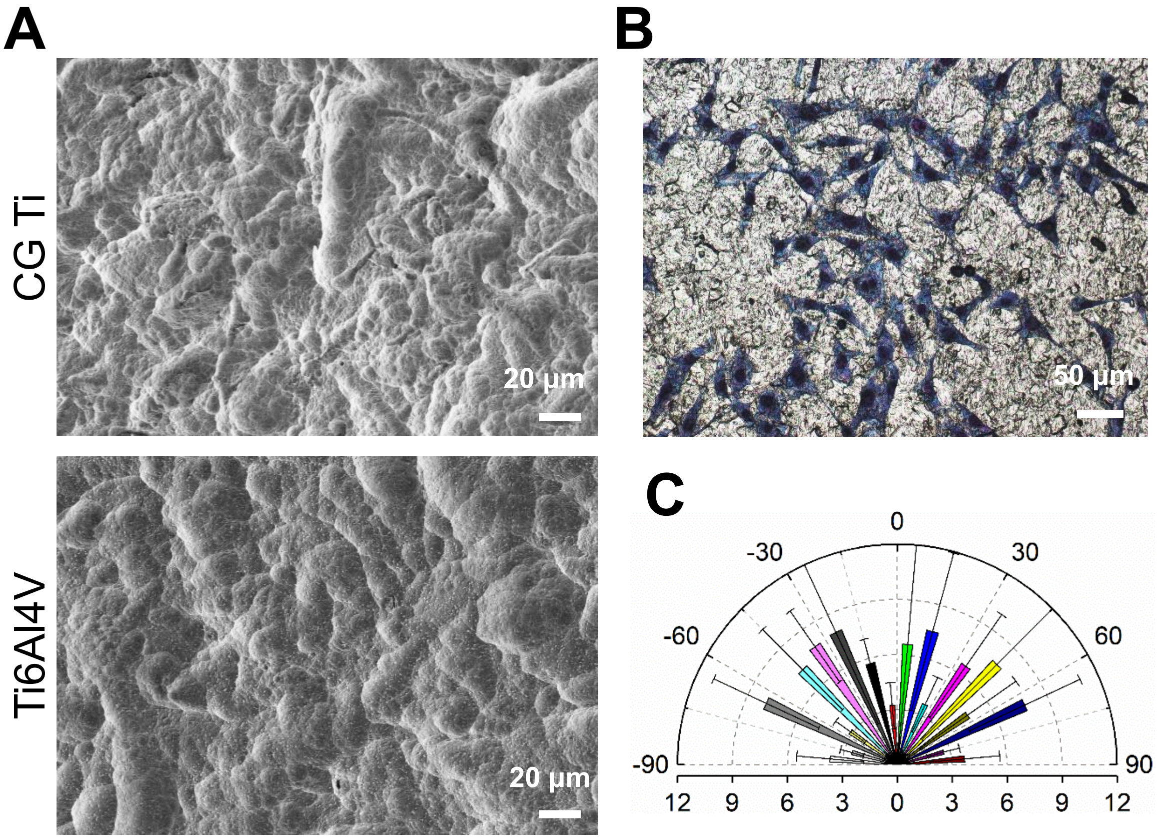

Figure S11. A) SEM image of Ti6Al4V and CG Ti after sandblasting and acid etching. B) Cell alignment and morphology observation by metallographic microscope on Ti6Al4V after 36 h spreading. C) Distribution of included angles (°) between cytoskeletons and arbitrary direction on Ti6Al4V. n = 80.

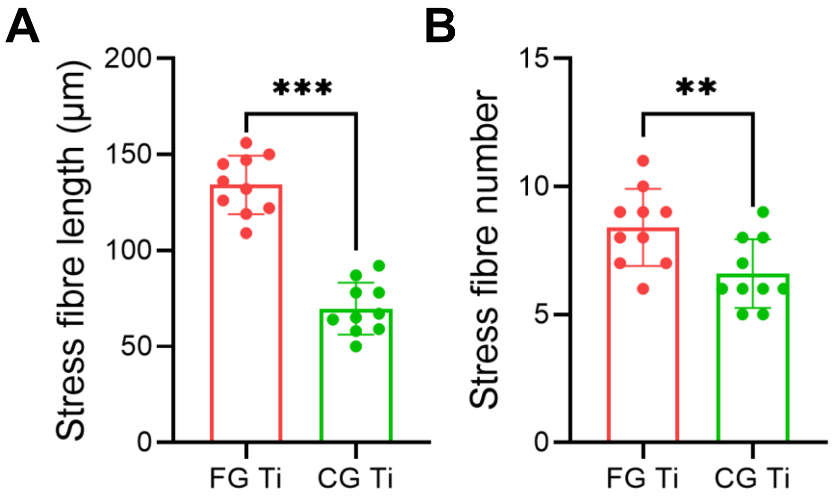

Figure S12. Stress fiber length A) and number B) of MC3T3E-1 cells on FG Ti and CG Ti after 36 h culture.

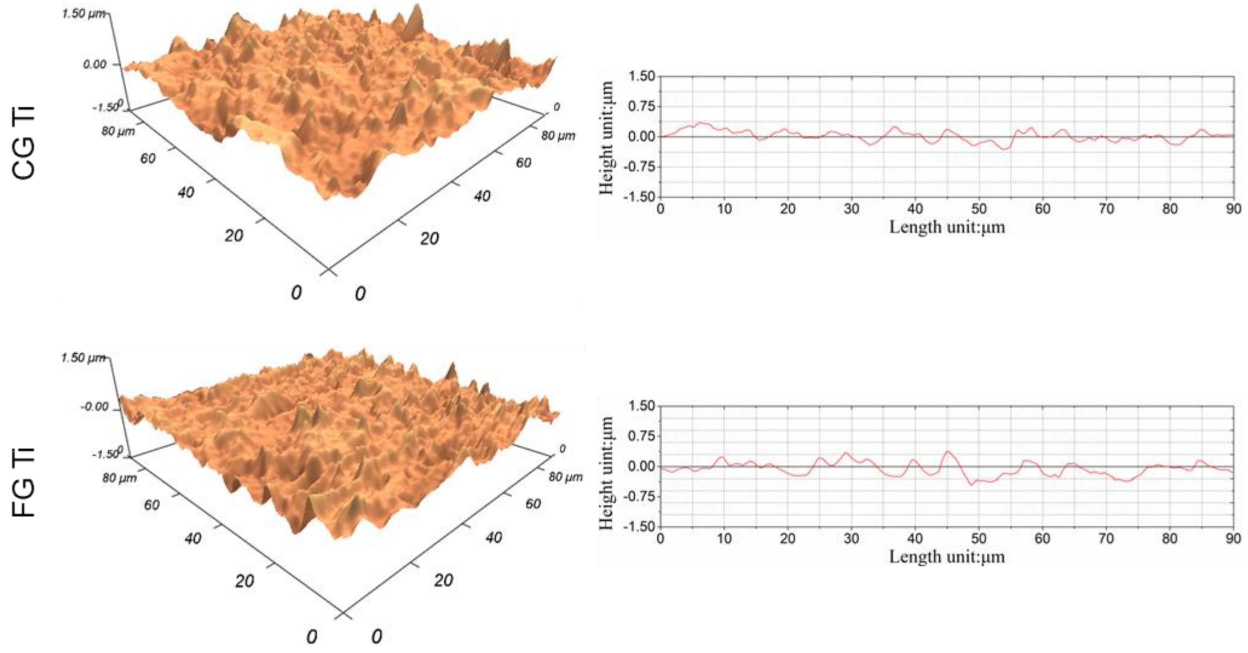

Figure S13. AFM analysis of FG Ti and CG Ti after acid-etching.

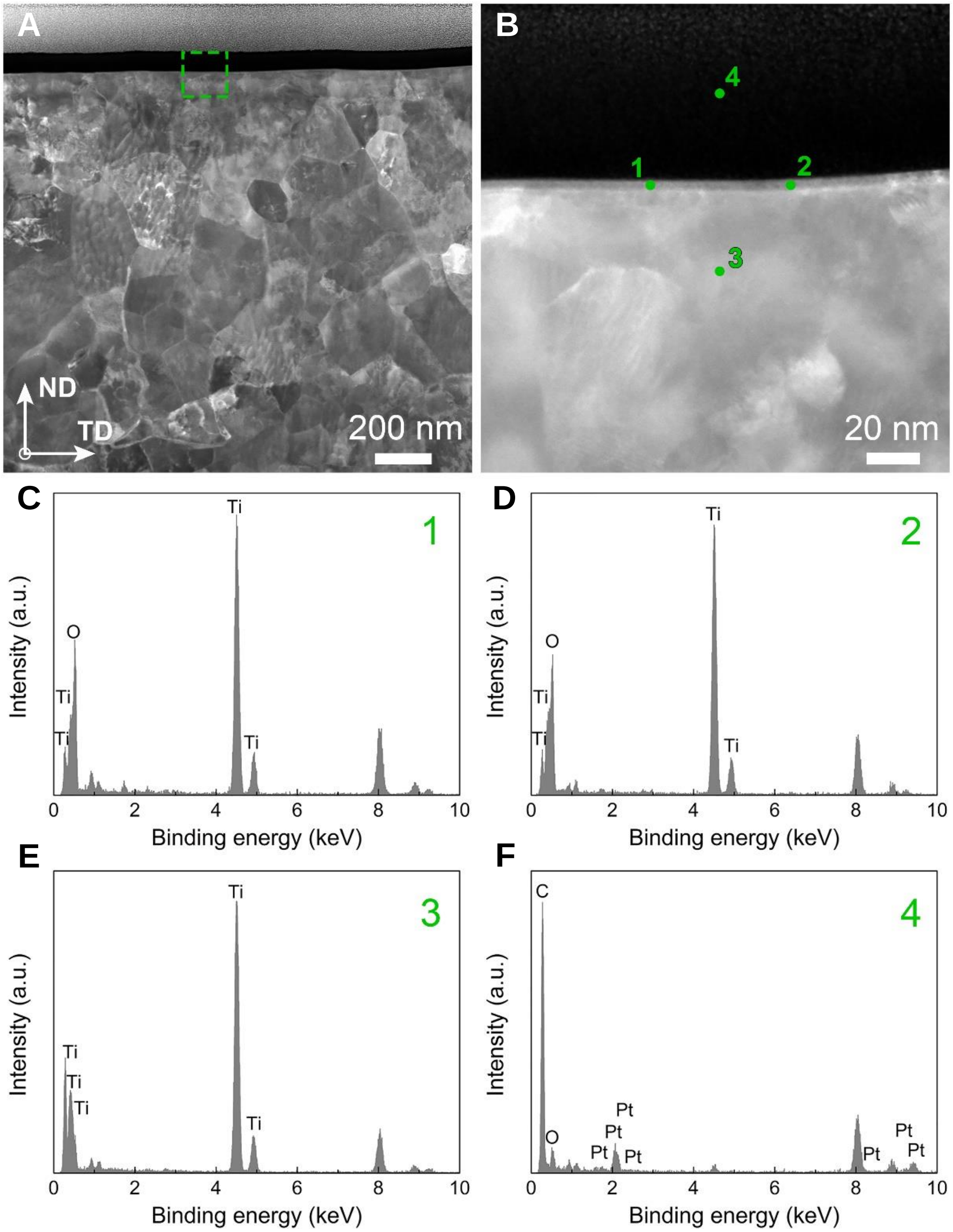

Figure S14. Determination of the chemical composition of the surface film. A) Typical scanning TEM micrograph showing an overview of the microstructure and surface film. B) Surface film at higher magnification by local magnification. C) to F) The energy dispersive spectroscopies of the 1, 2, 3, and 4 sites in b, respectively.

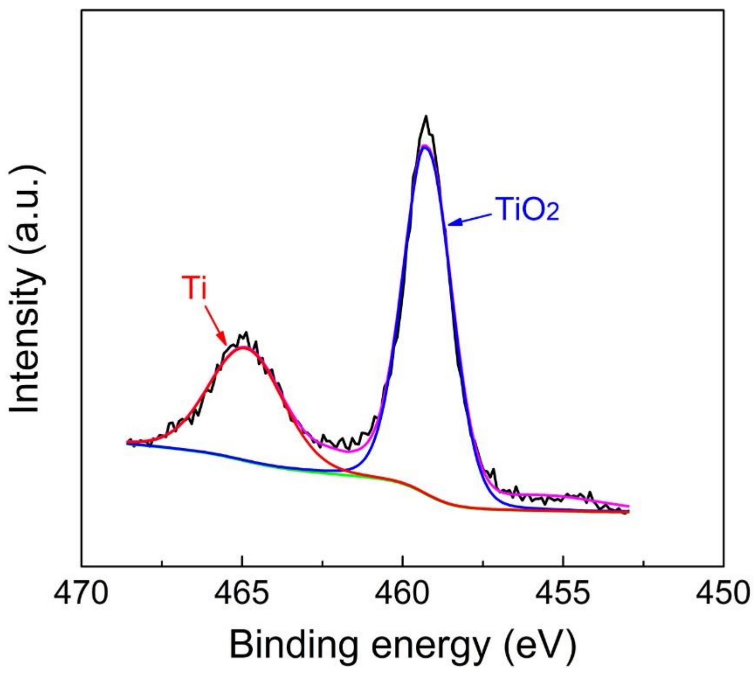

Figure S15. XPS spectra of oxide film formed on longitudinal section of FG Ti sample.

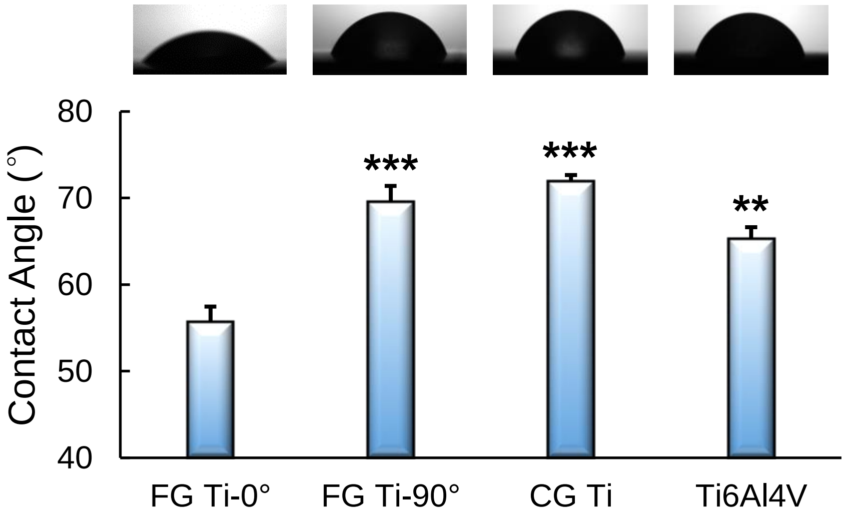

Figure S16. Optical images of water contact angle on various materials (FG Ti, CG Ti, and Ti6Al4V after acid etching) with different directions. (FG Ti-0°: parallel to RD, FG Ti-90°: vertical to RD). ***P* < 0.01, ****P* < 0.001 vs. FG Ti-0°.

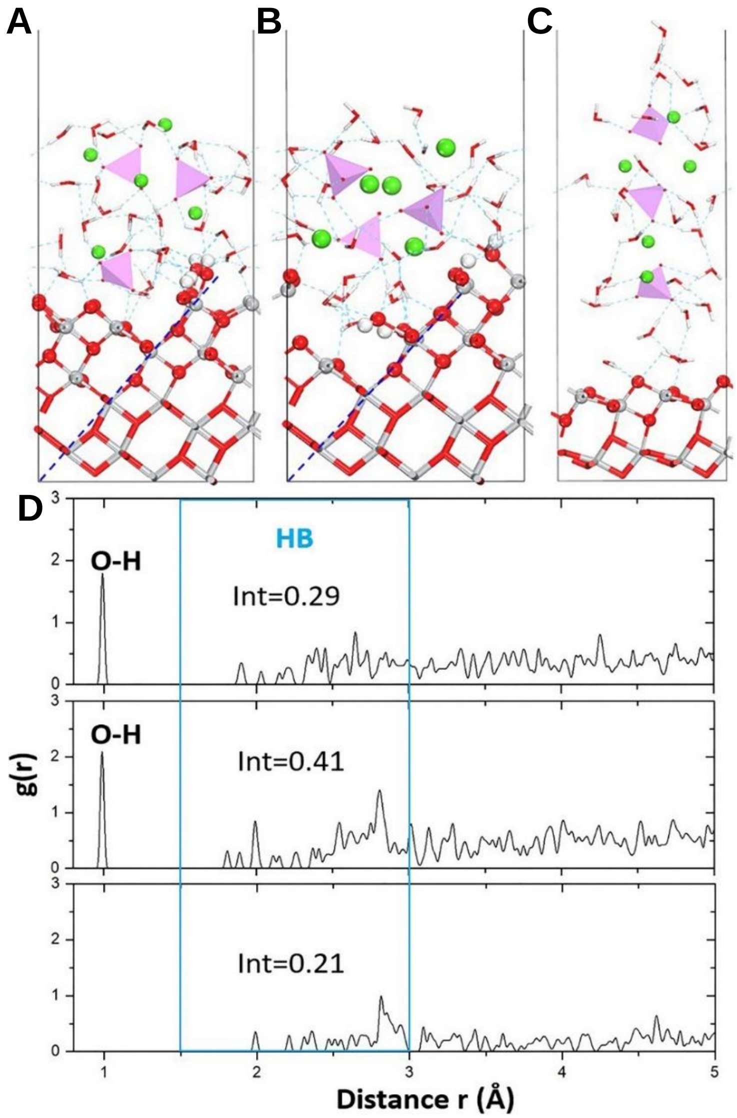

Figure S17. Computational simulations of HA/water over TiO_2 surfaces. A) to C) Fully optimized geometries for HA/water on ideal TiO_2(102), TiO_2(102)/TiO_2(001) combined surface and TiO_2(101) surface. D) Radical distribution function (RDF) g(r) for the interaction between TiO_2 substrate and HAP/water. Fixed bottom layers in TiO_2 have been shown as a thick stick, interface layers as ball-and-stick (Ti: grey; O: red), together with Ca^{2+} (green), H (white), and PO3-4 (pink tetrahedron). Hydrogen bonds (HBs) are shown as light blue lines and <001> direction was highlighted by dashed dark blue lines. RDF integration (Int) was carried out in the range of 1.5-3.0 Å (dominated by HBs). Interfacial O-H bonding associated with water dissociation is at ~1.0 Å, labeled as O-H.

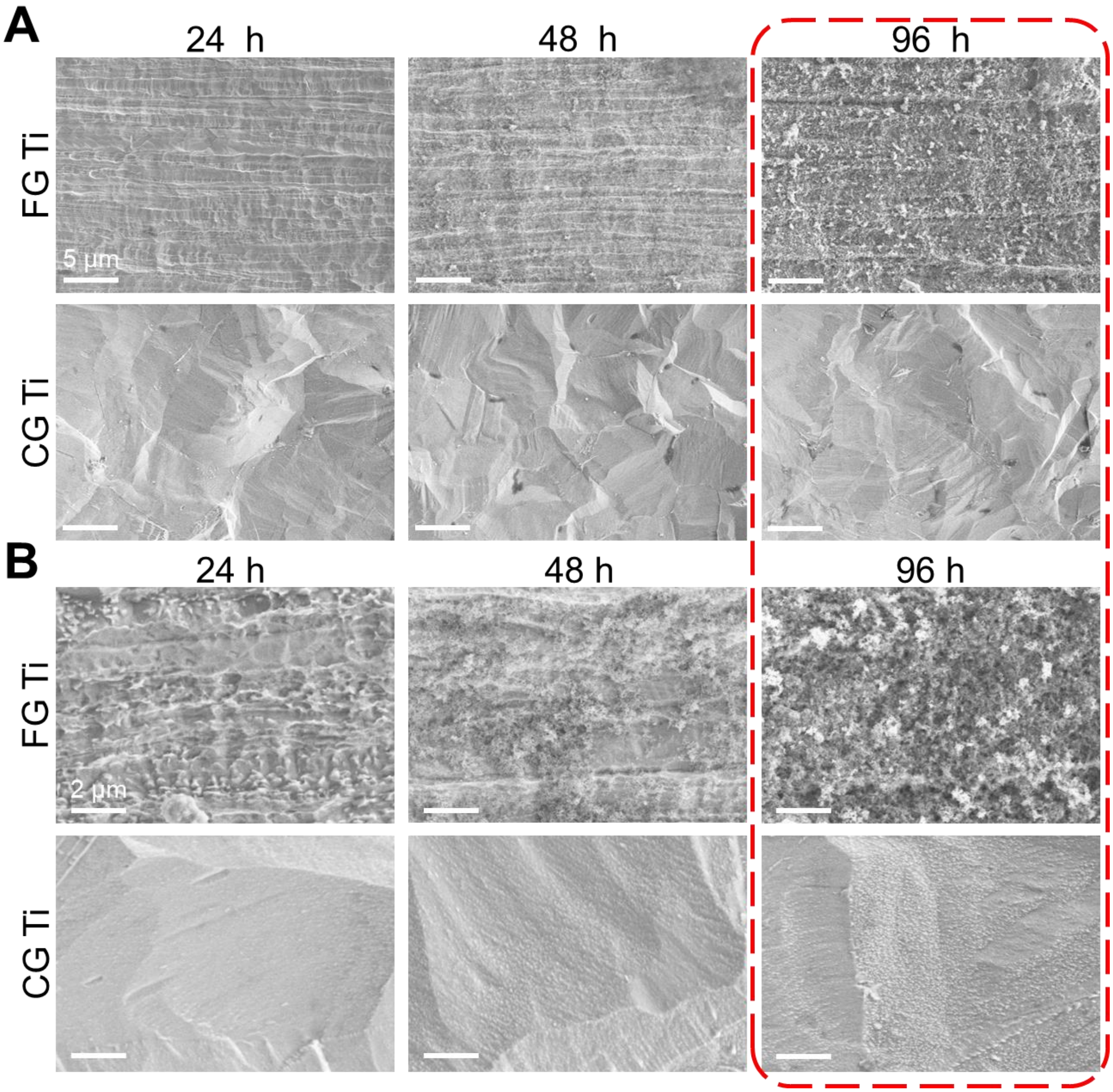

Figure S18. SEM images of FG Ti and CG Ti after soaking in SBF at 37 ^{o}C for 24, 48, and 96 h with different magnifications A) and B) (Scale bar = 5 or 2 μm).

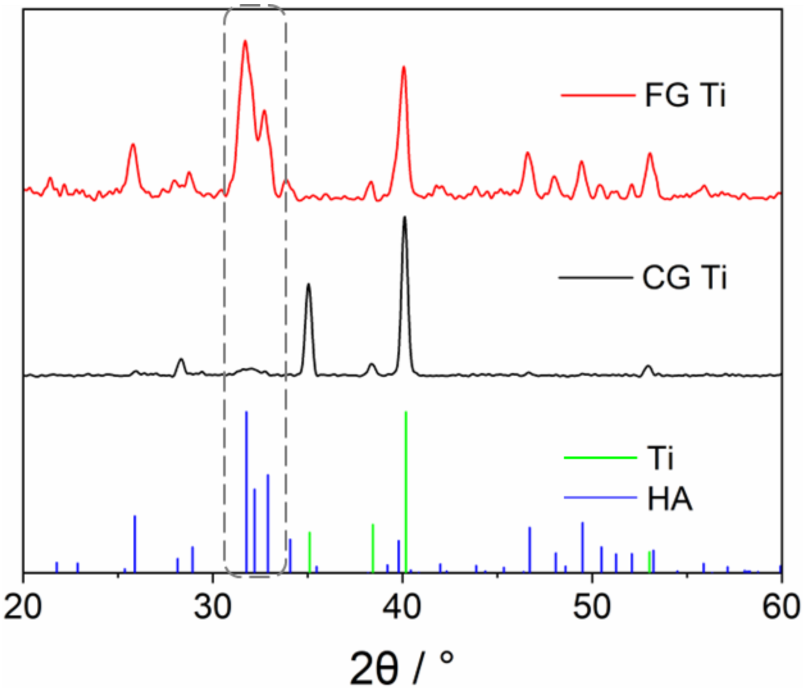

Figure S19. XRD analysis of FG Ti and CG Ti after soaking in SBF for 96 h at 37 ^{o}C.

Table S1. Chemical composition (wt. %) of studied pure Ti (Grade 2).

Fe	O	C	H	N	Ti
0.25	0.19	0.02	0.004	0.02	balance

Table S2. Tensile mechanical properties of FG Ti, CG Ti, and typical Ti6Al4V samples.

Mechanical properties	FG Ti	CG Ti	Pure Ti (Grade 2) (ISO 5832-3)	Pure Ti (Grade 2) (ASTM F67-13)	Ti6Al4V (ISO 5832-3)	Ti6Al4V (ASTM 1472-14)
$\sigma_{0.2}$ (MPa)	809 ± 11	313 ± 9	275	275	780	825
σ_b (MPa)	946 ± 14	452 ± 10	345	345	860	930
ε_u (%)	8.2 ± 0.6	12.0 ± 1.2	-	-	-	-
ε_f (%)	14.6 ± 1.0	26.4 ± 1.5	20	20	10	10

$\sigma_{0.2}$: yield strength; σ_b: ultimate strength; ε_u: uniform elongation; ε_f : elongation-to-fracture.

Table S3. The list of references in Figure S3.

Symbol	References
●	V. V. Stolyarov, L. Zeipper, B. Mingler, M. Zehetbauer, *Materials Science and Engineering: A* **2008**, *476*, 98
○	A. Mendes, A. M. Kliauga, M. Ferrante, V. L. Sordi, *IOP Conf. Ser.: Mater. Sci. Eng.* **2014**, *63*, 012161
◆	W. J. Kim, S. J. Yoo, J. B. Lee, *Scripta Materialia* **2010**, *62*, 451
⬢	G. Purcek, G. G. Yapici, I. Karaman, H. J. Maier, *Materials Science and Engineering: A* **2011**, *528*, 2303
⬟	S. V. Zherebtsov, G. S. Dyakonov, A. A. Salem, V. I. Sokolenko, G. A. Salishchev, S. L. Semiatin, *Acta Materialia* **2013**, *61*, 1167
●	J. L. Milner, F. Abu-Farha, C. Bunget, T. Kurfess, V. H. Hammond, *Materials Science and Engineering: A* **2013**, *561*, 109
⍰	Z. Li, L. Fu, B. Fu, A. Shan, *Materials Science and Engineering: A* **2012**, *558*, 309

Movie S1.

MC3T3-E1 cells migration on FG Ti.

Movie S2.

MC3T3-E1 cells migration on CG Ti.

Movie S3.

3-D reconstruction models based on the micro-CT of newly formed bone in the hole drilled for implantation around the screw of FG Ti at 3 weeks.

Movie S4.

3-D reconstruction models based on the micro-CT of newly formed bone in the hole drilled for implantation around the screw of FG Ti at 7 weeks.

Movie S5.

3-D reconstruction models based on the micro-CT of newly formed bone in the hole drilled for implantation around the screw of FG Ti at 12 weeks.

Movie S6.

3-D reconstruction models based on the micro-CT of newly formed bone in the hole drilled for implantation around the screw of CG Ti at 3 weeks.

Movie S7.

3-D reconstruction models based on the micro-CT of newly formed bone in the hole drilled for implantation around the screw of CG Ti at 7 weeks.

Movie S8.

3-D reconstruction models based on the micro-CT of newly formed bone in the hole drilled for implantation around the screw of CG Ti at 12 weeks.

Movie S9.

3-D reconstruction models based on the micro-CT of newly formed bone in the hole drilled for implantation around the screw of Ti6Al4V at 3 weeks.

Movie S10.

3-D reconstruction models based on the micro-CT of newly formed bone in the hole drilled for implantation around the screw of Ti6Al4V at 7 weeks.

Movie S11.

3-D reconstruction models based on the micro-CT of newly formed bone in the hole drilled for implantation around the screw of Ti6Al4V at 12 weeks.

Acronyms

Materials

CG Ti	Coarse-grained titanium
CMFDA	Chloromethyl fluorescein diacetate
DAPI	4',6-diamidino-2-phenylindole, blue-fluorescent DNA stain
FG Ti	Fibrous-grained titanium
FITC	Fluorescein isothiocyanate isomer
HCl	Hydrochloric acid
HF	Hydrofluoric acid
HNO_3	Nitric acid
ND	Normal direction of titanium rod
RD	Rolling direction of titanium rod
SBF	Simulated body fluid
TD	Transverse direction of titanium rod
Ti6Al4V	Titanium 6-aluminium 4-vanadium alloy

Methods

CLSM	Confocal laser scanning microscope
EBSD	Electron back-scattered diffraction
EDM	Electrical discharge machining
FIB	Focused ion beam
Micro-CT	Micro-computed tomography
SEM	Scanning electron microscope
SEN	Single-edge notch
TEM	Transmission electron microscope
XPS	X-ray photoelectron spectroscopy
XRD	X-ray diffraction
